# Metformin in severe exacerbations of chronic obstructive pulmonary disease: a randomised controlled trial

**DOI:** 10.1136/thoraxjnl-2015-208035

**Published:** 2016-02-25

**Authors:** Andrew W Hitchings, Dilys Lai, Paul W Jones, Emma H Baker

**Affiliations:** 1 Institute for Infection and Immunity, St George's, University of London, London, UK; 2 Chelsea and Westminster Hospital NHS Foundation Trust, London, UK

**Keywords:** COPD Exacerbations, COPD Pharmacology

## Abstract

**Background:**

Severe exacerbations of COPD are commonly associated with hyperglycaemia, which predicts adverse outcomes. Metformin is a well-established anti-hyperglycaemic agent in diabetes mellitus, possibly augmented with anti-inflammatory effects, but its effects in COPD are unknown. We investigated accelerated metformin therapy in severe COPD exacerbations, primarily to confirm or refute an anti-hyperglycaemic effect, and secondarily to explore its effects on inflammation and clinical outcome.

**Methods:**

This was a multicentre, randomised, double-blind, placebo-controlled trial testing accelerated metformin therapy in non-diabetic patients, aged ≥35 years, hospitalised for COPD exacerbations. Participants were assigned in a 2:1 ratio to 1 month of metformin therapy, escalated rapidly to 2 g/day, or matched placebo. The primary end point was mean in-hospital blood glucose concentration. Secondary end points included the concentrations of fructosamine and C reactive protein (CRP), and scores on the COPD Assessment Test and Exacerbations of Chronic Pulmonary Disease Tool.

**Results:**

52 participants (mean (±SD) age 67±9 years) were randomised (34 to metformin, 18 to placebo). All were included in the primary end point analysis. The mean blood glucose concentrations in the metformin and placebo groups were 7.1±0.9 and 8.0±3.3 mmol/L, respectively (difference −0.9 mmol/L, 95% CI −2.1 to +0.3; p=0.273). No significant between-group differences were observed on any of the secondary end points. Adverse reactions, particularly gastrointestinal effects, were more common in metformin-treated participants.

**Conclusion:**

Metformin did not ameliorate elevations in blood glucose concentration among non-diabetic patients admitted to hospital for COPD exacerbations, and had no detectable effect on CRP or clinical outcomes.

**Trial registration number:**

ISRCTN66148745 and NCT01247870.

**Key messages**

**What is the key question?**

- What are the effects of metformin in severe COPD exacerbations, particularly in relation to lowering blood glucose concentration?

**What is the bottom line?**

- In this randomised, placebo-controlled trial, metformin did not ameliorate in-hospital hyperglycaemia among non-diabetic patients admitted for COPD exacerbations nor did it affect the secondary end points of fructosamine, C reactive protein and patient-reported outcomes.

**Why read on?**

- Randomised controlled trials testing novel treatments for severe COPD exacerbations are urgently needed but exceptionally difficult and, as a result, are few and far between; in this trial we explored the anti-hyperglycaemic, anti-inflammatory and clinical effects of metformin in an acutely ill, inpatient COPD population and demonstrated that it is unlikely to offer benefit.

## Introduction

Exacerbations are major events for patients with COPD. They are associated with deconditioning, an increased risk of becoming housebound and reduced physical activity for weeks after onset of symptoms.^1^ Exacerbations often necessitate hospital admission, when they may be designated ‘severe’.^2^ Severe exacerbations are associated with a high risk of early mortality and a median survival of only 3.6 years.^3^ Specific medical treatment for most patients with severe COPD exacerbations comprises systemic corticosteroids, antibiotics and bronchodilators.^4^ These are at best modestly effective.^5^ ^6^ New strategies are urgently needed to improve outcomes of patients with severe COPD exacerbations.

Hyperglycaemia is an unexplored therapeutic target in COPD exacerbations. Elevated blood glucose concentrations occur in the majority of patients admitted to hospital for COPD exacerbations.^7–9^ The pathogenesis of this is probably multifactorial, including effects from systemic corticosteroid and inhaled β-agonist therapy,^10^ ^11^ hypoxia,^12^ acidosis,^13^ and stress-related increases in glucose-elevating hormones.^14^ ^15^ Elevated blood glucose concentration is associated with prolonged hospital stay and death, the risk of which increases by 7%–15% for each 1 mmol/L increment in blood glucose concentration,^7^ ^16^ ^17^ and with failure of non-invasive ventilation.^18^ Whether these associations are causal is unknown. However, given the well-established adverse effects of hyperglycaemia on oxidative stress, inflammation, immune function, endothelium and thrombosis,^19–21^ such a relationship is plausible and—subject to the availability of a suitable anti-hyperglycaemic agent—warrants investigation.

The ideal anti-hyperglycaemic agent would promote euglycaemia without causing hypoglycaemia, be widely available, inexpensive, free from serious adverse effects and amenable to administration both in hospital and at home. Metformin could fulfil these criteria, but its effects in acute medical illness, and COPD exacerbations in particular, are unknown. The present trial was conducted chiefly to test the anti-hyperglycaemic effects of metformin in severe COPD exacerbations, and secondarily to explore its safety, tolerability and effects on inflammation and clinical outcome.

## Methods

### Overview

This was a randomised, double-blind, placebo-controlled trial, conducted at nine acute NHS hospitals in the UK. The trial was designed, led and implemented entirely by academic investigators and NHS clinicians. It was approved by the South East Research Ethics Committee (reference 10/H1102/62) and implemented in accordance with Good Clinical Practice guidelines. It was publicly registered with ClinicalTrials.gov (NCT01247870) and the International Standard Randomised Controlled Trial Number (ISRCTN) Registry (ISRCTN66148745).

### Participants

**Figure 1:**
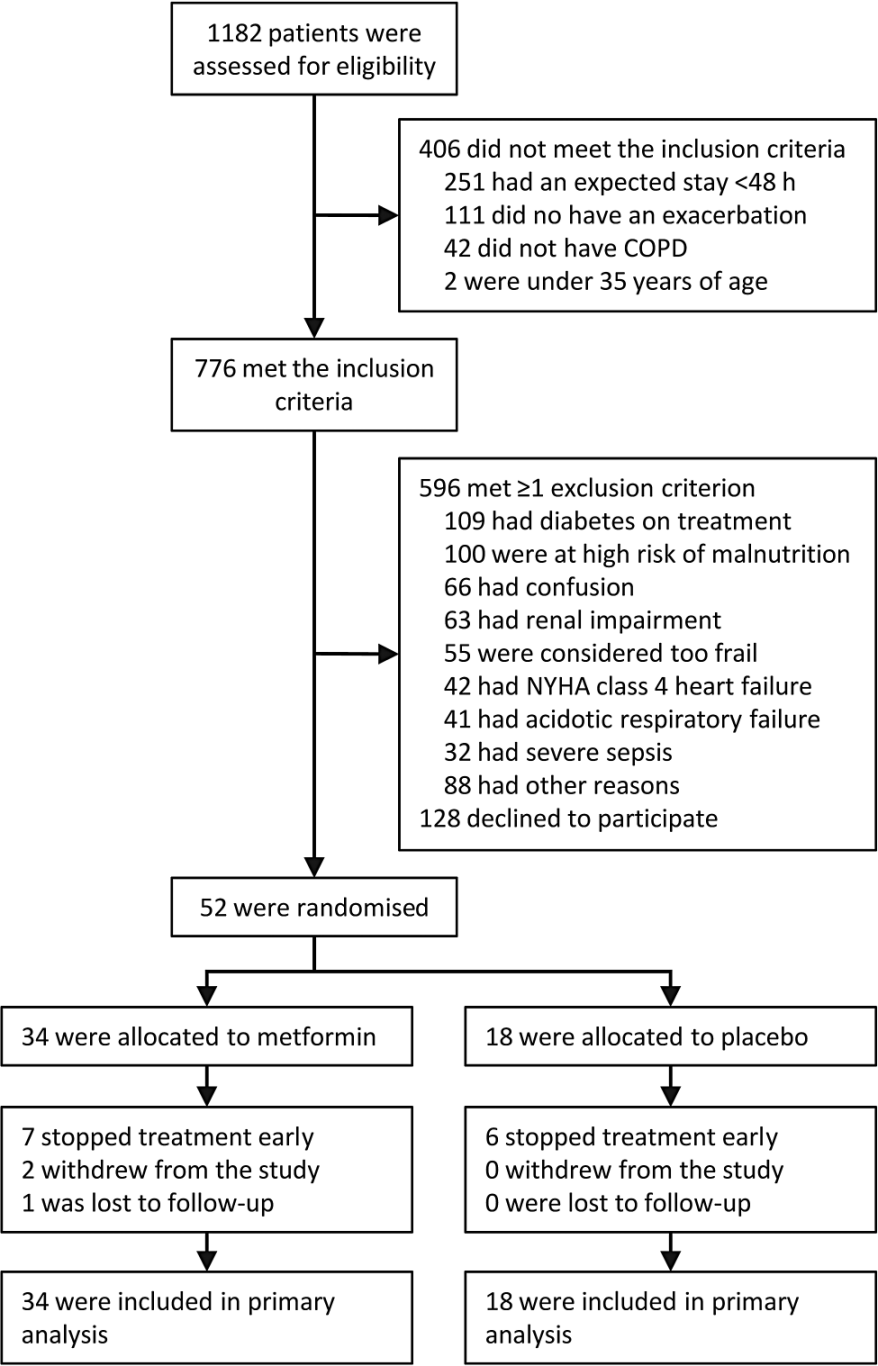
Flow diagram showing patient disposition. NYHA, New York Heart Association.

Patients were eligible to participate if they were aged ≥35 years, had COPD diagnosed by physician assessment and/or spirometry and were admitted to hospital for an acute exacerbation, with features as described by Anthonisen *et al*.^22^ To facilitate supervised initiation of study treatment, patients were also required to have an expected inpatient stay of ≥48 h. Exclusion criteria included pre-existing, pharmacologically treated diabetes mellitus, respiratory or metabolic acidaemia, risk factors for hypoglycaemia or lactate accumulation and other factors, such as confusion, which precluded trial participation (figure 1). Operational definitions for exclusion criteria are detailed in the trial protocol (see online supplementary data).

10.1136/thoraxjnl-2015-208035.supp1 Supplementary data

### Interventions

Study drugs were supplied by Sharp Clinical Services (formerly Bilcare GCS (Europe), Powys, UK), which over-encapsulated metformin 500 mg tablets and produced visually identical placebo capsules. The random allocation sequence, with a block size of six, was generated by the manufacturer and implemented through sequentially numbered containers. Neither participants nor investigators were aware of treatment assignment until after data lock. Emergency code breaks were administered independently of the trial investigators by staff of the St George's Healthcare NHS Trust Clinical Trials Pharmacy.

Participants were randomly assigned to metformin 500 mg capsules or matched placebo, starting at one capsule on day 1, incrementing to one capsule twice daily on day 2, two capsules in the morning and one in the evening on day 3 and two capsules twice daily from day 4 onwards. This was continued until a follow-up appointment at 1 month. Initially, the trial was designed with an equal allocation ratio. This was modified early in the recruitment phase to an active:placebo ratio of 2:1 on recommendation of a panel of external experts, on the basis that increased exposure to metformin would permit a more informed assessment of safety and tolerability. This modification was performed without revealing the treatment assignment for enrolled participants, and with a commensurate increase in sample size to preserve statistical power.

The protocol recommended that participants should receive oral prednisolone 30 mg/day for at least 7 days. Open-label oral anti-hyperglycaemic therapy was prohibited. All other care was at the discretion of the treating physicians.

### Assessments and end points

Scheduled study visits took place at baseline, hospital discharge and 1 month and telephone and postal contact with participants was made at 3 months. In addition, participants were reviewed regularly throughout the in-hospital phase to ascertain and encourage compliance with study procedures. They had access to 24 h telephone support post discharge. Capillary blood glucose concentration was measured seven times per day (pre-mealtime and 2 h post-mealtime and late evening) during the in-hospital phase, using point-of-care glucometers. Staff performing these measurements were blinded to treatment allocation but not necessarily to adverse events. Fructosamine and high-sensitivity C reactive protein (hsCRP) concentrations were measured at baseline, discharge and 1 month. The COPD Assessment Test (CAT) was also administered at these time points, and again at 3 months. The Exacerbations of Chronic Pulmonary Disease Tool (EXACT) was completed on paper every evening from randomisation for 1 month. Recurrent healthcare-utilisation events (systemic antibiotic and corticosteroid prescriptions and unplanned hospital admissions) were ascertained at 1 and 3 months. Plasma lactate concentration was monitored as a safety parameter, and details of adverse reactions and serious adverse events were sought at all contact opportunities.

The primary end point was mean in-hospital capillary glucose concentration, which has been found to be the most practical metric of hyperglycaemia-associated risk.^23^ Prespecified secondary end points were selected to supplement assessment of anti-hyperglycaemic effects (fructosamine), inflammation (hsCRP) and clinical recovery (CAT, EXACT and recurrent healthcare-utilisation events). Operational definitions for all end points, including time points for comparisons, were specified prospectively in the trial protocol.

### Sample size

The sample size was calculated to provide at least 90% power at the 5% significance level to detect a 1.5 mmol/L difference in the mean in-hospital capillary blood glucose concentration between groups, using an independent samples t test. This margin was selected to ensure the difference in blood glucose concentration observed between metformin and placebo in the settings of diabetes mellitus (1.9–3.6 mmol/L^24–26^) and burns injuries (2.3–4.5 mmol/L^27^ ^28^) would readily be captured. Based on local audit data, in which the SD of mean blood glucose concentration in inpatients with COPD was 1.5 mmol/L, we calculated that with equal allocation, 22 participants per group would be required. An initial target sample size of 23 per group was set to allow for up to two non-analysable participants. With the amendment to 2:1 allocation, described above, a minimum of 32 metformin-treated and 18 placebo-treated participants was required to preserve the a priori power specification. An actual target sample size of 46 and 23 metformin-treated and placebo-treated participants, respectively, was set arbitrarily (the original active treatment group was doubled) so as to offer additional data on the safety and tolerability of metformin in this context.

### Statistical analysis

The primary analysis was performed in accordance with the intention-to-treat principle. No interim analyses were planned or conducted. The prospectively defined statistical analysis plan specified the independent samples t test for the primary analysis of blood glucose concentration. Fructosamine was analysed by calculating the changes between baseline and follow-up, and comparing these between groups with independent samples t tests. CRP data were transformed to a natural log scale for analysis, with comparisons made between groups using the Wilcoxon signed-rank test, then anti-logged to permit the expression of changes over time as a percentage of the baseline value. Changes in CAT and EXACT scores were compared between groups with independent samples t tests. The prospectively defined time points for comparison were follow-up and 12 weeks for the CAT, and days 5, 10 and 28 for the EXACT (day 1 was defined by the first dose of the study drug). The significance of within-group changes in fructosamine, CRP, CAT and EXACT over the various study time points were evaluated using paired-samples t tests. Post hoc sensitivity analyses were conducted to test the robustness of findings to alternative analytical approaches. Statistical analyses were performed using SPSS (V.21.0; IBM Corp.).

## Results

### Participants

Between January 2011 and March 2014, we recruited 52 participants, all of whom were included in the primary analysis (figure 1). This exceeded the minimum number required for the prospectively defined power specification, but did not reach the larger target sample size set to enhance assessment of safety and tolerability. A decision was taken by the trial steering committee at this point, without reference to study data, to stop recruitment due to time and funding constraints, and imminent expiry of study drugs.

The groups were similar in baseline demographic and clinical characteristics (table 1) and concomitant treatment during the in-hospital phase (see online supplementary table S1). Spirometry, performed where possible at discharge and 1 month, was also similar between groups (see online supplementary table S2). Patients with diabetes mellitus already established on treatment were not eligible for inclusion, and no participant had pre-existing untreated diabetes mellitus. However, baseline haemoglobin A_1c_ (HbA_1c_) concentrations indicated a possible diagnosis of diabetes mellitus (≥48 mmol/mol) in 7 (18%) of the 39 patients in whom this measurement was obtained (median (IQR) 42 (40–44) and 42 (39–44) mmol/mol in metformin and placebo groups, respectively). Moreover, 41 participants (79%) had a blood glucose ≥10 mmol/L at least once during the in-hospital phase; in 29 (56%) this was ≥11.1 mmol/L.

**Table 1:** Baseline characteristics

Characteristic*	Metformin group (n=34)	Placebo group (n=18)	p Value
Age, year	69±10	65±8	0.258
Sex, M/F	21/13	11/7	0.963
Body mass index, kg/m^2^	26.0±5.9	25.6±6.1	0.834
Past medical history, n (%)			
Myocardial infarction	6 (18%)	1 (6%)	0.399
Heart failure	3 (9%)	1 (6%)	>0.99
Atrial fibrillation	4 (12%)	2 (11%)	>0.99
Cerebrovascular disease	3 (9%)	0 (0%)	0.543
Charlson comorbidity index	0.5 (0–1)	0.0 (0–1)	0.424
Smoking history, pack-years	54±52	54±21	0.961
Pulse, bpm	93±13	92±12	0.769
Systolic blood pressure, mm Hg	133±21	137±23	0.544
Diastolic blood pressure, mm Hg	75±10	80±13	0.133
Respiratory rate, breaths/min	19±2	19±3	0.705
Arterial blood gases			
pH	7.42±0.05	7.41±0.05	0.854
pCO_2_, kPa	5.5±1.2	5.6±1.1	0.649
pO_2_, kPa	9.2±2.5	9.4±2.2	0.824
Biochemistry			
Urea, mmol/L	5.7±1.7	5.4±2.3	0.693
Creatinine, μmol/L	73±17	72±23	0.861
Haemoglobin A_1c_, mmol/mol	42 (40–44)	42 (39–44)	0.821
C reactive protein, mg/L	14 (5–77)	17 (5–59)	0.840
Radiographic consolidation, n (%)	7 (21%)	3 (17%)	0.827

*Continuous data are presented as mean±SD or median (IQR), as applicable.

10.1136/thoraxjnl-2015-208035.supp2 Supplementary tables

### Primary end point

**Figure 2:**
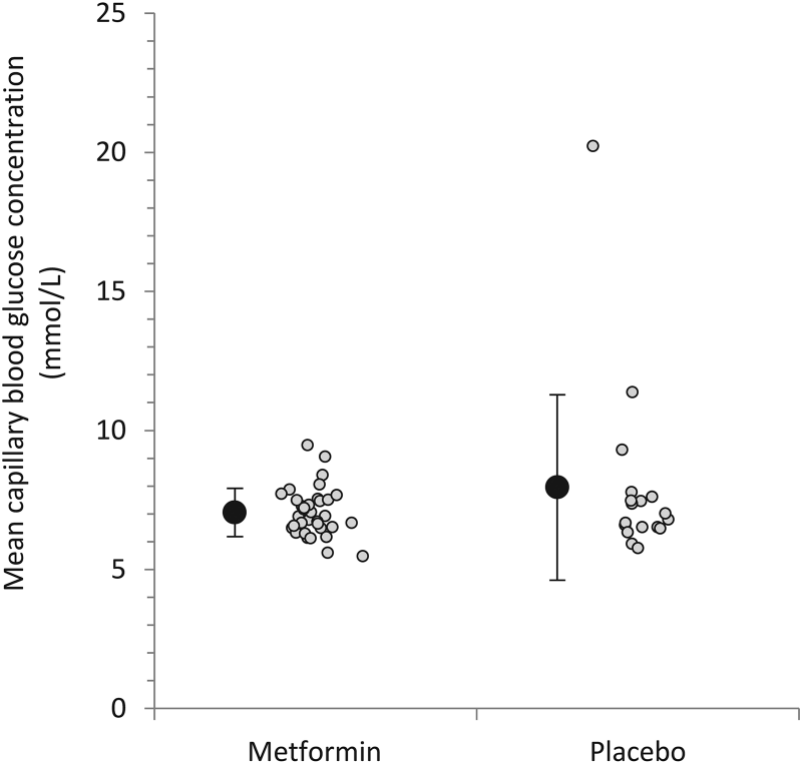
Plot showing mean in-hospital capillary blood glucose concentrations. Individual participant means are indicated by shaded circles, and group level means are indicated by solid circles with error bars denoting SD. The difference between the metformin and placebo groups was −0.9 mmol/L (95% CI −2.1 to +0.3; p=0.273). In a sensitivity analysis specified after data collection but before unblinding, in which an extreme outlier was excluded (mean blood glucose concentration 20.2 mmol/L), the difference between groups was −0.2 mmol/L (95% CI −0.8 to +0.45; p=0.566).

Mean capillary blood glucose measurements were derived from a mean (±SD) of 24±16 individual measurements per participant. The number of individual measurements did not differ between metformin and placebo groups (24±17 and 23±14, respectively; p=0.830). Mean in-hospital blood glucose concentration was 7.1±0.9 and 8.0±3.3 mmol/L, respectively, in the metformin and placebo groups (difference −0.9 mmol/L, 95% CI −2.1 to +0.3; p=0.273; figure 2).

### Sensitivity analyses

During data entry, it was noted that one participant had a mean glucose concentration of >10 SDs from the cohort mean. On the advice of the trial statistician, and without knowledge of this or any other participants’ treatment assignment, a sensitivity analysis excluding this participant was appended to the analysis plan. In this, the mean glucose concentration in the placebo group became 7.2±1.4 mmol/L, and the difference between metformin and placebo groups was −0.2 mmol/L (95% CI −0.8 to +0.4; p=0.566).

A post hoc mixed-effects analysis, incorporating trial site as a random factor, did not materially change the findings. Per protocol analysis, accounting for metformin suspension or early discontinuation, revealed no significant differences between groups. Substituting mean blood glucose with alternative summary metrics of glucose concentration similarly did not reveal any significant differences (see online supplementary table S3). In a subgroup analysis (see online supplementary table S4), a trend towards a glucose-lowering effect was more evident among participants whose mean day-1 blood glucose concentration was above the cohort median. However, the difference was not statistically significant (−2.3 mmol/L, 95% CI −6.1 to +1.5; p=0.201) and it was heavily influenced by the outlying observation (when excluded, difference −0.8, 95% CI −2.4 to +0.8; p=0.166). An analysis to investigate for the possibility of a delayed treatment effect identified no interaction between treatment allocation and study day (see online supplementary figure S1; p=0.832, analysis of variance). However, mean daily capillary blood glucose concentration fell over the first 7 inpatient study days (p<0.001).

10.1136/thoraxjnl-2015-208035.supp3 Supplementary figure Mean (standard deviation) capillary blood glucose concentrations for the first 7 inpatient study days.

### Secondary end points

#### Fructosamine

Overall, serum fructosamine concentration fell over the three time points examined in the study, but there were no significant differences between groups in this respect. In the metformin group, mean fructosamine concentration (expressed in µmol/L) was 225±39 at baseline, 210±23 at hospital discharge (p=0.031 for change from baseline) and 205±21 at 1 month (p=0.015). In the placebo group, the corresponding values at baseline, discharge and 1 month were 241±47, 245±50 (p=0.482) and 226±40 (p=0.175), respectively. The difference between metformin and placebo groups in respect of the change from baseline was −6 µmol/L (95% CI −22 to +10) at discharge and −1 µmol/L (−19 to +16) at 1 month.

#### C reactive protein

**Figure 3:**
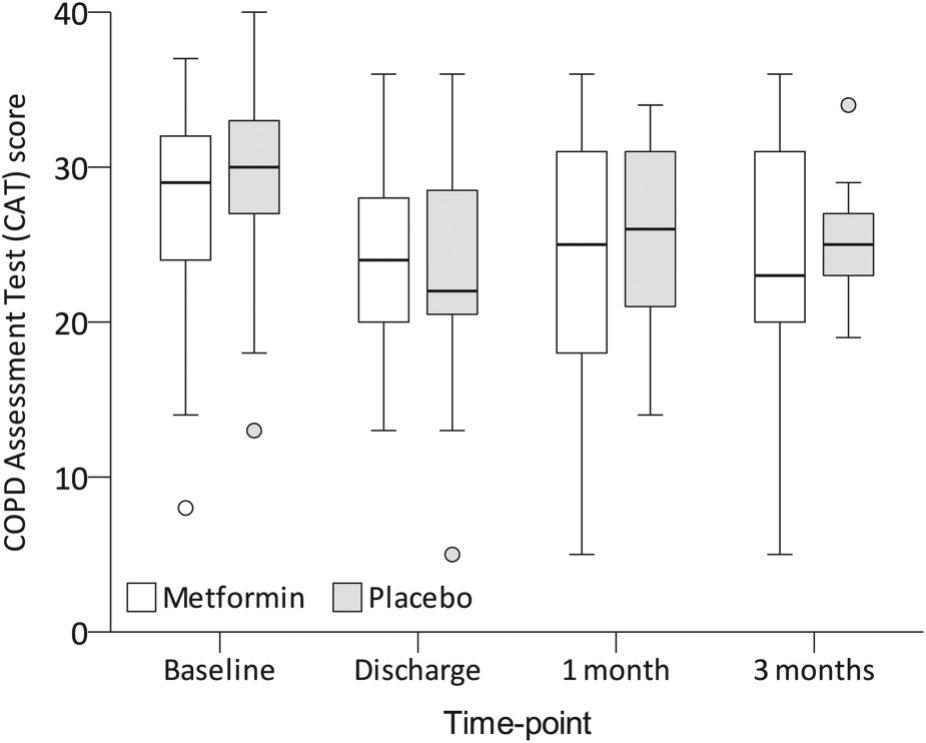
Box and whiskers plot showing high-sensitivity C reactive protein concentration. Medians are denoted by horizontal bars, IQRs by boxes and ranges by whiskers, save for any outliers which are denoted by circles. The lower limit of detection for the assay is indicated by a dashed line (0.3 mg/L).There were no significant differences in the absolute concentrations between the metformin and placebo groups at any time points.

The distribution of serum hsCRP measurements at baseline, discharge and 1 month is presented in figure 3. There were no differences between groups at any time point. Overall, expressed as percentages of the baseline value, hsCRP concentration fell to a median (IQR) of 30% (19%–52%) at discharge and 40% (17%–131%) at 1 month (p<0.001 and p=0.007 for changes from baseline to discharge and 1 month, respectively). However, there were no significant differences between groups in this respect (p=0.190 for the change between baseline and discharge, and 0.780 between baseline and 1 month).

#### Clinical end points

**Figure 4:**
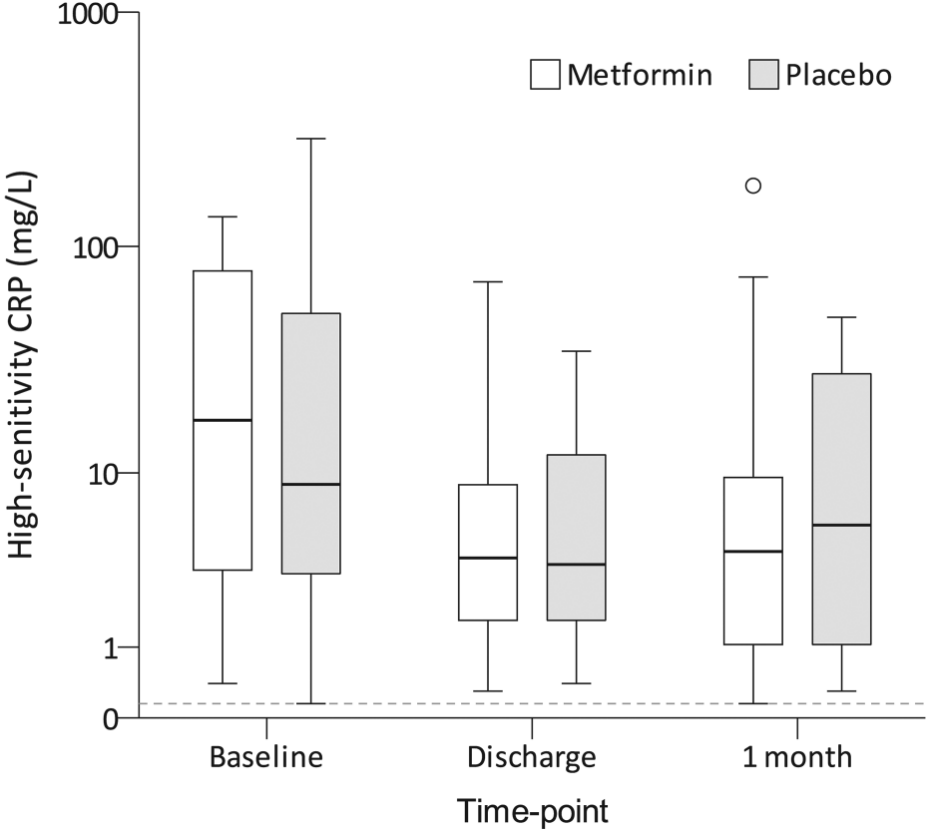
COPD Assessment Test (CAT) scores. CAT scores are on a 40-point scale, with higher scores indicating worse COPD-related health status. Medians are denoted by horizontal bars, IQRs by boxes and ranges by whiskers, save for any outliers which are indicated as circles. There were no significant differences between the groups at any time point.

Overall, mean CAT scores were 28±6 points at baseline, falling to 24±7 at discharge (p<0.001 for change from baseline), 25±7 at 1 month (p=0.001) and 25±7 at 3 months (p=0.063). This did not differ between groups (figure 4). Measured from baseline, the proportion of participants with at least a 5-point improvement was 50% at discharge, 43% at 1 month and 41% at 3 months; this was similar between groups.

**Figure 5:**
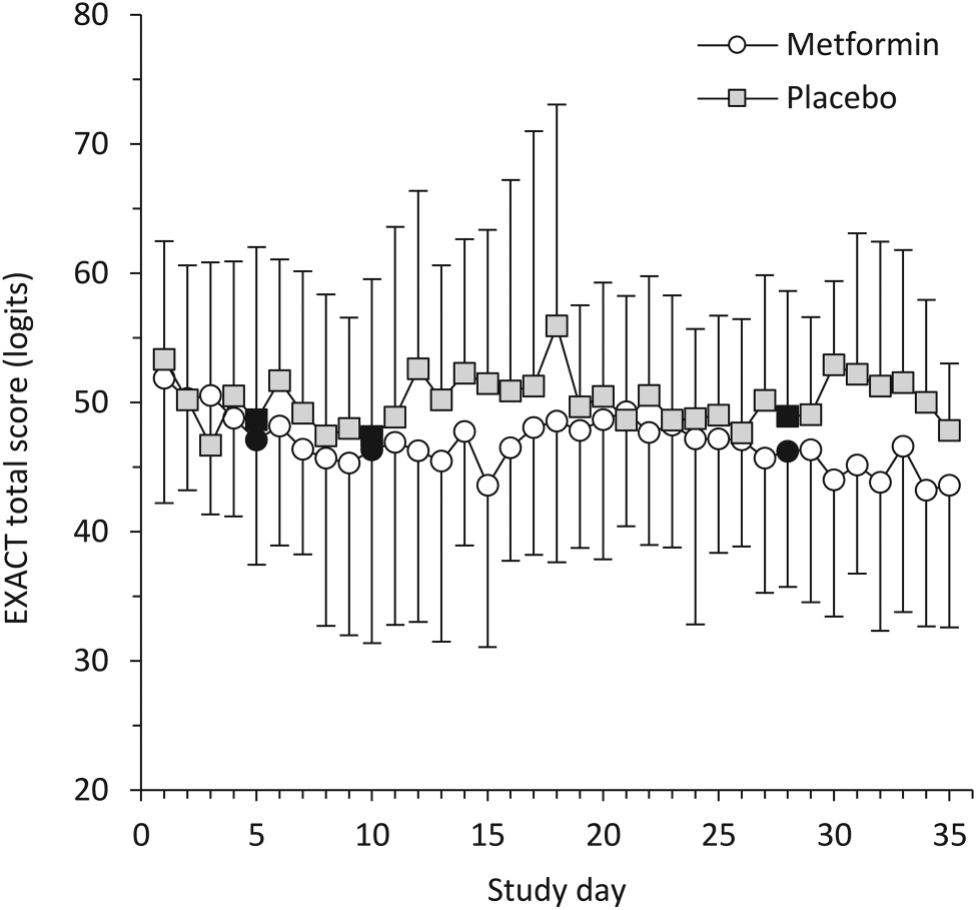
Exacerbations of Chronic Pulmonary Disease Tool (EXACT) scores. EXACT scores are on a 100-point logit scale, with higher scores indicating worse symptoms. Error bars indicate SDs. There were no significant differences between the groups at the three prospectively defined time points for comparison (indicated by solid markers). Day 1 was defined as the first day on which the study medication was administered.

EXACT scores were measured daily, but days 5, 10 and 28 were the predefined time points for comparisons (day 1 was defined by the first study drug dose). Overall, the EXACT score was 52±9 at baseline, falling to 48±11 on day 5 (p=0.041 for change from baseline), 48±11 on day 10 (p=0.054) and 47±10 (p=0.042) on day 28. The groups were similar in this respect (figure 5).

Taking the study cohort as a whole, in the 3 months from index admission, 23 participants (44%) experienced 37 further hospital admission episodes, 23 (44%) required 30 further systemic corticosteroid prescriptions and 27 (52%) required 39 further antibiotic prescriptions. The median times to any event (metformin group 46 days vs placebo group 40 days, p=0.682); readmission (76 vs 50 days, p=0.814) and systemic corticosteroid and/or antibiotic prescription (49 vs 63 days, p=0.769) were similar between groups. Median length of stay was also similar, at 6.5 days (4.3–11.5) from admission in the metformin group and 7.0 days (4.8–9.3) in the placebo group (p=0.968). Measured from study entry, corresponding figures were 5.0 days (3.0–8.8) and 5.0 days (4.0–7.3) (p=0.737).

### Safety and tolerability

Twelve participants (23%) experienced at least one hypoglycaemic event, defined as blood glucose concentration of ≤3.9 mmol/L, during the in-hospital phase. There was no significant difference between groups in this respect, with six patients (18%) in the metformin group and six (33%) in the placebo group experiencing an event (p=0.202). Mean lactate concentration was 2.0 mmol/L in both groups (mean difference 0.0, 95% CI −0.3 to +0.3 mmol/L, p=0.879). There were no cases of lactic acidosis, defined prospectively as a lactate concentration of ≥5 mmol/L with acidaemia, in either group.

There were 69 adverse events in the metformin group and 25 in the placebo group (event rates 2.0 and 1.4 per participant, respectively; p=0.149; table 2). Related, non-serious adverse events were more common in the metformin group. This was largely due to an excess of gastrointestinal symptoms, which affected 24 patients (71%) in the metformin group and five patients (28%) in the placebo group (relative risk 2.54, 95% CI 1.17 to 5.52, p=0.003). Changes to study treatment (dose reduction, suspension or early discontinuation) due to an adverse event were required for 13 patients (38%) in the metformin group and three patients (17%) in the placebo group (relative risk 2.29, 95% CI 0.75 to 7.02, p=0.109).

**Table 2:** Adverse events

	Frequency (events per participant)		
Event classification	Metformin (n=34)	Placebo (n=18)	p Value
Relationship with study treatment considered at least possible (adverse reactions)			
Non-serious	31 (0.91)	6 (0.33)	0.011
Serious*	3 (0.09)	–	0.199
Required or prolonged hospital admission	3 (0.09)	–	
Relationship with study treatment considered unlikely			
Non-serious	23 (0.68)	16 (0.89)	0.417
Serious*	12 (0.35)	3 (0.17)	0.366
Resulted in death	3 (0.09)	–	
Resulted in significant disability or incapacity	–	1 (0.06)	
Required or prolonged hospital admission	9 (0.26)	2 (0.11)	

This table presents the frequency of adverse events in each group and, due to the unequal group sizes and the potential for participants to experience more than one adverse event, the event rates per participant. Classification by relatedness and seriousness is based on the designations assigned by local investigators according to the Good Clinical Practice adverse event definitions.

*Individual events could have more than one reason for a ‘serious’ designation; in these instances, the event is classified according to the most severe reason specified.

Three participants (6%) died during the study period; all deaths were due to respiratory deterioration and none were judged to be related to the study.

## Discussion

We conducted a multicentre, randomised, double-blind, placebo-controlled trial to explore the effects of metformin in patients admitted to hospital for exacerbations of COPD. We found no difference in the primary end point of mean in-hospital capillary glucose concentration, despite participants having a clear tendency towards hyperglycaemia. There were no differences in any of the prespecified secondary end points, including fructosamine, hsCRP, patient-reported outcome measures (CAT and EXACT) and recurrent healthcare-utilisation events. Adverse reactions, particularly gastrointestinal effects, were more common in metformin-treated participants. Lactate concentration did not differ between groups and there were no cases of lactic acidosis.

Our finding that metformin did not ameliorate blood glucose elevations in COPD exacerbations contrasts with its well-established anti-hyperglycaemic effects in diabetes.^29^ This may be due to the differing pathophysiology of hyperglycaemia in COPD exacerbations. In this context it is notable that, despite metformin having been available for well over half a century, few studies have explored its glucose-lowering effect in acute illnesses. The only studies to address this question directly were two small trials in a burns unit,^27^ ^28^ in which a glucose-lowering effect was observed, and an open-label study in critically ill patients,^30^ in which metformin appeared to be insulin-sparing. The present study differs from these in several important respects. First and foremost, it was conducted in the distinct context of COPD exacerbations, where the pathogenesis of hyperglycaemia may be different to that in burn injuries and critical illness. Second, the sample size was larger, and with its multicentre, double-blind design, the methodology was arguably more robust. Third, the participants in the earlier trials had a greater propensity to hyperglycaemia than those in the present trial. Using the placebo groups as comparators, the mean glucose concentrations in the earlier trials were in the range of 10.2–11.3 mmol/L,^27^ ^28^ ^30^ as compared with 8.0 mmol/L in the present study. Given that metformin acts to reduce blood glucose towards, but not below, the normal range,^29^ it is plausible that there was less scope in the present study for metformin to exert a detectable, clinically meaningful, glucose-lowering effect. Supporting this, a post hoc analysis suggested a trend towards a more pronounced glucose-lowering effect in participants with above-median baseline glucose concentrations.

Inflammation is central to the pathophysiology of COPD. It is evident both in the lungs and the serum, and markers of inflammation, notably CRP, rise during exacerbations and fall with recovery.^31^ Studies in type 2 diabetes^32^ ^33^ and polycystic ovarian syndrome^34^ ^35^ suggest that metformin may have anti-inflammatory effects, evident particularly as a fall in the serum CRP concentration. This finding was not reproduced in the present study. CRP was a secondary end point which did not contribute to the sample size determination and, as such, the negative results do not confirm the absence of an effect. It is also possible that a longer treatment period may be necessary, given that the positive studies lasted months to years, whereas this and other studies,^36^ ^37^ of shorter duration, reported negative results.

The study was not powered to detect changes in clinical end points. However, these were explored to assist in planning future effectiveness studies, if indicated. Overall, scores on the patient-reported outcome instruments improved over the follow-up period, with the trends in favour of metformin, but no significant differences were identified between groups. Likewise, there were trends in favour of metformin, but no significant differences, in the risk of recurrent healthcare-utilisation events. Integrating these non-significant trends with the negative mechanistic findings on anti-hyperglycaemic and anti-inflammatory effects, our interpretation is that a meaningful short-term clinical benefit with metformin in this population is unlikely.

This study has some important limitations. Despite a recruitment period lasting over 3 years and involving nine sites—a considerable escalation over what had originally been envisaged—we were unable to enrol as many participants as intended. In large part this was due to the high prevalence of serious acute and chronic comorbidities among the patients screened. Our experience illustrates the challenges faced when conducting research in this setting, as is reflected in the scarcity of clinical trials testing novel treatments for COPD exacerbations. However, the sample size was sufficient to meet the prospectively defined power specification; its main impact was in constraining our assessment of tolerability and safety. The study cohort was also highly selected, which inevitably brings into question the generalisability of the findings. However, the tendency to hyperglycaemia seen in the present study was similar to that observed in two other inpatient COPD cohorts,^7^ ^18^ and the mean baseline HbA_1c_ (43 mmol/mol or 6.1%) was the same as that seen in another similar cohort.^8^

Capillary blood glucose concentration was selected as the primary outcome because it is the glycaemic metric most extensively studied in COPD exacerbations, and which has been clearly associated with adverse outcomes.^7^ ^8^ ^16^ ^17^ However, point-of-care glucometers are subject to greater analytical error than laboratory analysis of venous blood, which may reduce measurement precision. This is mitigated by the fact that, being less burdensome and costly, capillary glucose can be measured more frequently and thus better capture biological fluctuations. Moreover, the negative primary end point data were supported by similar findings on the supplementary glycaemic metric of fructosamine, which reflects average blood glucose concentration over the preceding 2–3 weeks.^38^

In conclusion, in this randomised controlled trial conducted in non-diabetic patients admitted to hospital for COPD exacerbations, who were prone to elevated blood glucose concentrations, metformin had no detectable anti-hyperglycaemic effect and did not significantly alter CRP or clinical end points, although these secondary end points were not powered. Collectively, the findings suggest that as an acute treatment for COPD exacerbations in non-diabetic individuals, metformin is unlikely to be beneficial.

## References

[1] Donaldson GC, Wilkinson TM, Hurst JR, et al Exacerbations and time spent outdoors in chronic obstructive pulmonary disease. Am J Respir Crit Care Med 2005;171:446–52. 10.1164/rccm.200408-1054OC 15579723

[2] Wedzicha JA, Seemungal TA COPD exacerbations: defining their cause and prevention. Lancet 2007;370:786–96. 10.1016/S0140-6736(07)61382-8 17765528 PMC7134993

[3] Suissa S, Dell'Aniello S, Ernst P Long-term natural history of chronic obstructive pulmonary disease: severe exacerbations and mortality. Thorax 2012;67:957–63. 10.1136/thoraxjnl-2011-201518 22684094 PMC3505864

[4] National Clinical Guideline Centre. Chronic obstructive pulmonary disease: management of chronic obstructive pulmonary disease in adults in primary and secondary care. NICE clinical guideline CG101 2010 http://www.nice.org.uk/guidance/cg101/evidence/full-guideline-134519581

[5] Walters JA, Gibson PG, Wood-Baker R, et al Systemic corticosteroids for acute exacerbations of chronic obstructive pulmonary disease. Cochrane Database Syst Rev 2009;(1):CD001288 10.1002/14651858.CD001288.pub3 19160195

[6] Vollenweider DJ, Jarrett H, Steurer-Stey CA, et al Antibiotics for exacerbations of chronic obstructive pulmonary disease. Cochrane Database Syst Rev 2012;(12):CD010257 10.1002/14651858.CD010257 23235687

[7] Baker EH, Janaway CH, Philips BJ, et al Hyperglycaemia is associated with poor outcomes in patients admitted to hospital with acute exacerbations of chronic obstructive pulmonary disease. Thorax 2006;61:284–9. 10.1136/thx.2005.051029 16449265 PMC2104606

[8] Koskela HO, Salonen PH, Niskanen L Hyperglycaemia during exacerbations of asthma and chronic obstructive pulmonary disease. Clin Respir J 2013;7:382–9. 10.1111/crj.12020 23902130

[9] Burt MG, Roberts GW, Aguilar-Loza NR, et al Continuous monitoring of circadian glycemic patterns in patients receiving prednisolone for COPD. J Clin Endocrinol Metab 2011;96:1789–96. 10.1210/jc.2010-2729 21411550

[10] Slatore CG, Bryson CL, Au DH The association of inhaled corticosteroid use with serum glucose concentration in a large cohort. Am J Med 2009;122:472–8. 10.1016/j.amjmed.2008.09.048 19375557

[11] Smith AP, Banks J, Buchanan K, et al Mechanisms of abnormal glucose metabolism during the treatment of acute severe asthma. Q J Med 1992;82:71–80. 1438670

[12] Louis M, Punjabi NM Effects of acute intermittent hypoxia on glucose metabolism in awake healthy volunteers. J Appl Physiol 2009;106:1538–44. 10.1152/japplphysiol.91523.2008 19265062 PMC2681331

[13] Adrogué HJ, Chap Z, Okuda Y, et al Acidosis-induced glucose intolerance is not prevented by adrenergic blockade. Am J Physiol 1988;255 (Pt 1):E812–23. 3144181 10.1152/ajpendo.1988.255.6.E812

[14] Fujiwara T, Cherrington AD, Neal DN, et al Role of cortisol in the metabolic response to stress hormone infusion in the conscious dog. Metabolism 1996;45:571–8. 10.1016/S0026-0495(96)90026-8 8622599

[15] McGuinness OP, Shau V, Benson EM, et al Role of epinephrine and norepinephrine in the metabolic response to stress hormone infusion in the conscious dog. Am J Physiol 1997;273:E674–81. 9357794 10.1152/ajpendo.1997.273.4.E674

[16] Burt MG, Roberts GW, Aguilar-Loza NR, et al Relationship between glycaemia and length of hospital stay during an acute exacerbation of chronic obstructive pulmonary disease. Intern Med J 2013;43:721–4. 10.1111/imj.12157 23745995

[17] Koskela HO, Salonen PH, Romppanen J, et al A history of diabetes but not hyperglycaemia during exacerbation of obstructive lung disease has impact on long-term mortality: a prospective, observational cohort study. BMJ Open 2015;5:e006794 10.1136/bmjopen-2014-006794 PMC4316436 25633287

[18] Chakrabarti B, Angus RM, Agarwal S, et al Hyperglycaemia as a predictor of outcome during non-invasive ventilation in decompensated COPD. Thorax 2009;64:857–62. 10.1136/thx.2008.106989 19454410

[19] Evans JL, Goldfine ID, Maddux BA, et al Oxidative stress and stress-activated signaling pathways: a unifying hypothesis of type 2 diabetes. Endocr Rev 2002;23:599–622. 10.1210/er.2001-0039 12372842

[20] Yu WK, Li WQ, Li N, et al Influence of acute hyperglycemia in human sepsis on inflammatory cytokine and counterregulatory hormone concentrations. World J Gastroenterol 2003;9:1824–7. 10.3748/wjg.v9.i8.1824 12918129 PMC4611552

[21] Chittari MV, McTernan P, Bawazeer N, et al Impact of acute hyperglycaemia on endothelial function and retinal vascular reactivity in patients with Type 2 diabetes. Diabet Med 2011;28:450–4. 10.1111/j.1464-5491.2010.03223.x 21204962

[22] Anthonisen NR, Manfreda J, Warren CP, et al Antibiotic therapy in exacerbations of chronic obstructive pulmonary disease. Ann Intern Med 1987;106:196–204. 10.7326/0003-4819-106-2-196 3492164

[23] Kosiborod M, Inzucchi SE, Krumholz HM, et al Glucometrics in patients hospitalized with acute myocardial infarction: defining the optimal outcomes-based measure of risk. Circulation 2008;117:1018–27. 10.1161/CIRCULATIONAHA.107.740498 18268145

[24] Stumvoll M, Nurjhan N, Perriello G, et al Metabolic effects of metformin in non-insulin-dependent diabetes mellitus. N Engl J Med 1995;333:550–4. 10.1056/NEJM199508313330903 7623903

[25] [No authors listed] Effect of intensive blood-glucose control with metformin on complications in overweight patients with type 2 diabetes (UKPDS 34). UK Prospective Diabetes Study (UKPDS) Group. Lancet 1998;352:854–65. 10.1016/S0140-6736(98)07037-8 9742977

[26] Johansen K Efficacy of metformin in the treatment of NIDDM. Meta-analysis. Diabetes Care 1999;22:33–7. 10.2337/diacare.22.1.33 10333900

[27] Gore DC, Wolf SE, Sanford A, et al Influence of metformin on glucose intolerance and muscle catabolism following severe burn injury. Ann Surg 2005;241:334–42. 10.1097/01.sla.0000152013.23032.d1 15650645 PMC1356920

[28] Gore DC, Wolf SE, Herndon DN, et al Metformin blunts stress-induced hyperglycemia after thermal injury. J Trauma 2003;54:555–61. 10.1097/01.TA.0000026990.32856.58 12634538

[29] Bailey CJ, Turner RC Metformin. N Engl J Med 1996;334:574–9. 10.1056/NEJM199602293340906 8569826

[30] Mojtahedzadeh M, Rouini MR, Kajbaf F, et al Advantage of adjunct metformin and insulin therapy in the management of glycemia in critically ill patients. Evidence for nonoccurrence of lactic acidosis and needing to parenteral metformin. Arch Med Sci 2008;4:174–81.

[31] Perera WR, Hurst JR, Wilkinson TM, et al Inflammatory changes, recovery and recurrence at COPD exacerbation. Eur Respir J 2007;29:527–34. 10.1183/09031936.00092506 17107990

[32] Haffner S, Temprosa M, Crandall J, et al Intensive lifestyle intervention or metformin on inflammation and coagulation in participants with impaired glucose tolerance. Diabetes 2005;54:1566–72. 10.2337/diabetes.54.5.1566 15855347 PMC1314967

[33] Lund SS, Tarnow L, Stehouwer CD, et al Impact of metformin versus repaglinide on non-glycaemic cardiovascular risk markers related to inflammation and endothelial dysfunction in non-obese patients with type 2 diabetes. Eur J Endocrinol 2008;158:631–41. 10.1530/EJE-07-0815 18426821

[34] Tsilchorozidou T, Mohamed-Ali V, Conway GS Determinants of interleukin-6 and C-reactive protein vary in polycystic ovary syndrome, as do effects of short- and long-term metformin therapy. Horm Res 2009;71:148–54. 10.1159/000197871 19188739

[35] Diamanti-Kandarakis E, Paterakis T, Alexandraki K, et al Indices of low-grade chronic inflammation in polycystic ovary syndrome and the beneficial effect of metformin. Hum Reprod 2006;21:1426–31. 10.1093/humrep/del003 16497699

[36] Eriksson A, Attvall S, Bonnier M, et al Short-term effects of metformin in type 2 diabetes. Diabetes Obes Metab 2007;9:330–6. 10.1111/j.1463-1326.2006.00611.x 17391159

[37] De Jager J, Kooy A, Lehert P, et al Effects of short-term treatment with metformin on markers of endothelial function and inflammatory activity in type 2 diabetes mellitus: a randomized, placebo-controlled trial. J Intern Med 2005;257:100–9. 10.1111/j.1365-2796.2004.01420.x 15606381

[38] Juraschek SP, Steffes MW, Miller ER III, et al Alternative markers of hyperglycemia and risk of diabetes. Diabetes Care 2012;35:2265–70. 10.2337/dc12-0787 22875225 PMC3476908

