# Supplementary material for: Metformin in severe exacerbations of chronic obstructive pulmonary disease: a randomised controlled trial
Source: Thorax. 2016 Feb 25;71(7):587–93. doi: 10.1136/thoraxjnl-2015-208035 (PMC4941151; doi:10.1136/thoraxjnl-2015-208035)
Supplement: Supplementary data [file thoraxjnl-2015-208035supp.pdf]

CLINICAL STUDY PROTOCOL

A randomised, double-blind, placebo-controlled trial of metformin in chronic obstructive pulmonary disease (COPD) exacerbations: a pilot study

Study short name: Metformin in COPD

Sponsor's R&D Registration Number: 10.0086
EudraCT number: 2010-020818-28

CHIEF INVESTIGATOR:
Prof Emma Baker
Professor of Clinical Pharmacology
Division of Biomedical Sciences (Mail Point J1A)
St George's, University of London
Cranmer Terrace
London, SW17 0RE

Phone: 020 8725 5383
Email: ebaker@sgul.ac.uk
Secretary: 0208 725 0164
Fax: 020 8725 2993
Pager: Aircall SG 648

SPONSOR REPRESENTATIVE:
Debbie Rolfe
St George's Joint Research Office (JRO)
Hunter Wing, Ground Floor
Cranmer Terrace
London SW17 0RE
Phone: 020 8725 5013
Email: drolfe@sgul.ac.uk
Fax: 020 8725 0794

Information in this protocol is confidential and should not be disclosed, other than to those directly involved in the execution or the ethical/regulatory review of the study, without written authorisation from St George's Joint Research Office (JRO) or its affiliates.

Signature Page and Statement

The Chief Investigator (CI) and the JRO have discussed this protocol. The investigators agree to perform the investigations and to abide by this protocol except in case of medical emergency (Section 11.11) or where departures from it are mutually agreed in writing.

The investigator agrees to conduct the trial in compliance with the protocol, GCP and UK Regulations for CTIMPs, the Data Protection Act (1998), the Trust Information Governance Policy (or other local equivalent), the Research Governance Framework (2005), the Sponsor's SOPs, and other regulatory requirements as appropriate.

This protocol has been written in accordance to the Sponsor's procedure outlined in the Standard Operating Procedure (SOP) identified as: JRODOC001 CTIMP Protocol Template

Chief Investigator
Prof Emma Baker
Professor of Clinical Pharmacology
St George's, University of London

Signature Date

Sponsor Representative
Debbie Rolfe
Regulatory Assurance Manager
Joint Research & Enterprise Office
St George's University of London
St George's Healthcare NHS Trust

Signature Date

Contents

1 List of abbreviations 6
2 Study personnel 8
3 Study synopsis 10
4 Introduction 12
4.1 Background 12
4.1.1 Chronic obstructive pulmonary disease (COPD) 12
4.1.2 COPD exacerbation 12
4.1.3 Stress hyperglycaemia 12
4.1.4 Management of hyperglycaemia in patients with critical illness 13
4.1.5 Management of hyperglycaemia in non-critically ill hospitalised patients 13
4.2 Investigational Medicinal Product (IMP) 13
4.3 Metformin—pre-clinical data 14
4.3.1 Pharmacokinetics 14
4.3.2 Mechanism of action 14
4.3.3 Anti-inflammatory and anti-oxidant effects 14
4.4 Metformin—clinical data 15
4.4.1 Effects and uses in type 2 diabetes mellitus 15
4.4.2 Anti-hyperglyaemic effects in acute illness 15
4.5 Potential benefits of metformin therapy in the context of COPD exacerbation 16
4.5.1 Anti-hyperglycaemic action 16
4.5.2 Reduced requirement for insulin 16
4.5.3 Favourable changes in oxidative stress–antioxidant balance 16
4.5.4 Improving steroid sensitivity 16
4.6 Assessment and management of risks 17
4.6.1 Minor side effects 17
4.6.2 Hypoglycaemia 17
4.6.3 Lactic acidosis 18
5 Study objectives 20
5.1 Primary objective 20
5.2 Secondary objectives 20
6 Trial design 20
6.1 Overall design 20
6.2 Study population 20
6.3 Dosage regimen and rationale 20
6.4 Treatment period 21
6.5 Concomitant treatment 21
6.6 Schematic of trial design 21
7 Inclusion and exclusion criteria 23
7.1 Inclusion criteria 23
7.2 Exclusion criteria 23
8 Recruitment 25
8.1 Subject recruitment process 25
9 Study procedures and schedule of assessments 26

9.1 Informed consent ... 26
9.2 Randomisation procedure ... 26
9.3 Emergency unblinding ... 27
9.4 Screening assessments ... 27
9.5 Treatment procedure ... 27
9.6 Summary flow chart of study assessments ... 28
9.7 Procedure for missed and duplicated study assessments ... 29
9.8 Methods ... 29
9.8.1 Laboratory procedures ... 29
9.9 Definition of end of trial ... 29
9.10 Discontinuation/withdrawal of participants and stopping rules ... 30
9.10.1 Subject withdrawal procedure ... 30
9.10.2 Treatment suspension and stopping rules ... 30
10 Name and description of all drugs used in the trial ... 33
10.1 Name, description and source of each IMP and placebo ... 33
10.2 Accountability procedures for the investigation product, including the placebo ... 33
10.3 Route of administration, dosage, dosage regimen, and treatment period(s) of the IMPs 34
10.4 Dosage modifications ... 34
10.5 Assessment of compliance ... 34
10.6 Post-trial IMP arrangements ... 34
10.7 Name and description of each NIMP ... 35
11 Pharmacovigilance ... 35
11.1 Definitions ... 35
11.2 Recording Adverse Events (AEs) ... 36
11.3 Procedures for recording and reporting SAEs ... 36
11.4 Protocol Defined Events (PDEs) ... 38
11.5 Notification of deaths ... 38
11.6 Reporting of SUSARs ... 38
11.7 The type and duration of the follow-up of subjects after AEs ... 39
11.8 Developmental Safety Update Reports (DSURs) ... 39
11.9 Annual Progress Reports (APRs) ... 39
11.10 Pregnancy ... 40
11.11 Reporting urgent safety measures ... 40
11.12 Notification of serious breaches to GCP and/or the protocol ... 40
12 Data management and quality assurance ... 42
12.1 Confidentiality ... 42
12.2 Data collection tool ... 42
12.3 Data handling and analysis ... 42
13 Archiving arrangements ... 43
14 Statistical methods ... 43
14.1 Statistical input in trial design ... 43
14.2 Endpoints ... 43
14.2.1 Primary endpoint ... 43
14.2.2 Secondary endpoints ... 43

14.2.3 Feasibility, safety and tolerability assessment 44
14.3 Sample size and recruitment 45
14.3.1 Sample size calculation 45
14.3.2 Planned recruitment rate 45
14.4 Statistical analysis plan 45
14.4.1 Summary of baseline data and flow of patients 45
14.4.2 Primary endpoint analysis 46
14.4.3 Secondary endpoint analysis 46
14.4.4 Sub-group analyses 46
14.4.5 Sensitivity and other planned analyses 47
14.5 Randomisation 47
14.6 Interim analysis 47
14.7 Other statistical considerations 47
15 Committees in involved in the trial 47
16 Direct access to source data 48
17 Ethics and regulatory requirements 48
18 Monitoring plan for the trial 48
19 Finance 49
20 Insurance and indemnity 49
21 Publication policy 49
22 Statement of compliance 49
23 References 51

1 List of abbreviations

AE	Adverse Event
APTR	Activated Partial Thromboplastin Ratio
AR	Adverse Reaction
ASR	Annual Safety Report
CA	Competent Authority
CAT	COPD Assessment Test
CI	Chief Investigator
COPD	Chronic Obstructive Pulmonary Disease
CRF	Case Report Form
CRP	C-reactive protein
CRO	Contract Research Organisation
CTA	Clinical Trial Authorisation
CTIMP	Clinical Trial of Investigational Medicinal Product
DMC	Data Monitoring Committee
EC	European Commission
e-GFR	Estimated Glomerular Filtration Rate
EMEA	European Medicines Agency
EU	European Union
EUCTD	European Clinical Trials Directive
EudraCT	European Clinical Trials Database
EudraVIGILANCE	European database for Pharmacovigilance
EXACT	EXAcerbations of Chronic Pulmonary Disease Tool
FEV_1	Forced Expiratory Volume in 1 second
g	Gram
GAfREC	Governance Arrangements for NHS Research Ethics
GCP	Good Clinical Practice
GMP	Good Manufacturing Practice
HbA1c	Haemoglobin A1c
IB	Investigator Brochure
ICF	Informed Consent Form
IMP	Investigational Medicinal Product
IMPD	Investigational Medicinal Product Dossier
ISF	Investigator Site File
ISRCTN	International Standard Randomised
L	Litre
MA	Marketing Authorisation
Main REC	Main Research Ethics Committee
MHRA	Medicines and Healthcare products Regulatory Agency
mg	Milligram
mL	Millilitre
mol	Mole
MRC	Medical Research Council
MS	Member State
MUST	Malnutrition Universal Screening Tool

NHS R&D	National Health Service Research & Development
PI	Principal Investigator
PIL	Participant Information Leaflet
QA	Quality Assurance
QC	Quality Control
QP	Qualified Person for release of trial drug
RCT	Randomised Control Trial
REC	Research Ethics Committee
SAR	Serious Adverse Reaction
SAE	Serious Adverse Event
SDV	Source Document Verification
SmPC	Summary of Product Characteristics
SOP	Standard Operating Procedure
SpO2	Oxygen saturation as measured by pulse oximetry
SSA	Site Specific Assessment
SSAR	Suspected Serious Adverse Reaction
SUSAR	Suspected Unexpected Serious Adverse Reaction
TMG	Trial Management Group
TSC	Trial Steering Committee
UO	Urine Output

2 Study personnel

Chief Investigator (CI): **Prof Emma Baker**
Professor of Clinical Pharmacology
Division of Biomedical Sciences (Mail Point J1A)
St George's, University of London
Email: ebaker@sgul.ac.uk
Phone: 020 8725 5383
Fax: 020 8725 2993

Statistician: **Dr Jan Poloniecki**
Senior Lecturer in Medical Statistics
Community Health Sciences
St George's, University of London
E-mail: sggp300@sgul.ac.uk
Phone: 020 8725 2795
Fax: 020 8725 3584

Routine local laboratories: Local on-site (NHS) laboratories

Pharmacy: **Nia Al-Samarrai**
Lead Research Pharmacist
Pharmacy
Atkinson Morley Wing
St George' Healthcare NHS Trust
Blackshaw Road, London
SW17 0QT
E-mail: Nia.Alsamarrai@stgeorges.nhs.uk
Phone: 020 8725 1294
Fax: 020 8725 4167

Co-investigator **Dr Andrew Hitchings**
Specialist Registrar in Clinical PharmacologyDivision of Biomedical Sciences (Mail Point J1A)
St George's, University of London
E-mail: ahitchin@sgul.ac.uk
Phone: 020 8725 0205
Fax: 020 8725 2993

Co-investigator **Dr Dilys Lai**
Consultant Physician
Chelsea and Westminster Hospital
369 Fulham Road
London
SW10 9NH

**Co-investigator**	**Prof Paul Jones**<br>Professor of Respiratory Medicine, Head of Division of Clinical Science<br>St George's, University of London<br>E-mail: pjones@sgul.ac.uk<br>Phone: 020 8725 5371<br>Fax: 020 8725 5955
**Co-investigator**	**Dr Claire Wells**<br>Clinical Research Fellow<br>St George's, University of London<br>E-mail: cwells@sgul.ac.uk<br>Phone: 020 8725 5380<br>Fax: 020 8725 2993
**Co-investigator**	**Dr Natasha Patel**<br>Consultant in Diabetes and Acute Medicine<br>Thomas Addison Unit<br>St George' Healthcare NHS Trust<br>Blackshaw Road, London<br>SW17 0QT<br>E-mail: Natasha.patel@stgeorges.nhs.uk<br>Phone: 020 8725 6089
**Co-investigator**	**Dr Anjali Balasanthiran**<br>Clinical Research Fellow<br>Collaboration for Leadership in Applied Health Research and Care<br>Chelsea and Westminster Hospital<br>369 Fulham Road<br>London<br>SW10 9NH
**Co-investigator**	**Cielito Caneja**<br>Respiratory Nurse Specialist<br>Chelsea and Westminster Hospital<br>369 Fulham Road<br>London<br>SW10 9NH

3 Study synopsis

**Study number:**	10.0086
**Study drug:**	Metformin
**Title of the study:**	A randomised, double-blind, placebo-controlled trial of metformin in chronic obstructive pulmonary disease exacerbations: a pilot study
**Chief Investigator:**	Prof Emma Baker<br>Professor of Clinical Pharmacology<br>Division of Biomedical Sciences<br>St George's, University of London<br>Cranmer Terrace, London, SW17 0RE
**Study centres:**	• St George's Healthcare NHS Trust<br>• Chelsea and Westminster Hospital NHS Foundation Trust<br>• Conquest Hospital, East Sussex Healthcare NHS Trust<br>• Freeman Hospital, Newcastle upon Tyne NHS Foundation Trust<br>• Kings Mill Hospital, Sherwood Forests NHS Foundation Trust<br>• University Hospital of North Tees, North Tees & Hartlepool NHS Foundation Trust<br>• Blackpool Victoria Hospital, Blackpool, Fylde and Wyre Hospitals NHS Foundation Trust<br>• Royal Preston Hospital, Lancashire Teaching Hospitals NHS Foundation Trust<br>• Royal Lancaster Infirmary, University Hospitals of Morecambe Bay
**Study period:**	01/07/2010 – 01/07/2014
**Clinical phase:**	03/01/2011 – 31/03/2014
**Primary Objective:**	To determine whether, in patients hospitalised for COPD exacerbations and receiving conventional treatment, the addition of metformin, as compared with placebo, ameliorates hyperglycaemia.
**Secondary Objective:**	To determine whether, in patients hospitalised for COPD exacerbations and receiving conventional treatment, the addition of metformin, as compared with placebo, produces effects on clinical outcome, and measures of inflammation, steroid sensitivity, and airflow obstruction, which merit further study.
**Study population:** Patients hospitalised for COPD exacerbations and expected to stay in the hospital for ≥ 48 hours. The target sample size is 69 patients.	
**Methodology:** Randomised, double-blind, placebo-controlled trial. Patients will be randomised in a 2:1 ratio to metformin or matched placebo. Treatment will be commenced at randomisation and continued until follow-up at day 28–35.	

Main Inclusion and Exclusion criteria:

Inclusion criteria:

1. Diagnosis of COPD
2. Hospitalisation for exacerbation of COPD
3. Age ≥35 years
4. Expected to remain in hospital for ≥48 hours.

Exclusion criteria:

1. Prior diagnosis of diabetes mellitus requiring insulin or oral hypoglycaemic therapy
2. Hypersensitivity to metformin hydrochloride or to any of the excipients
3. Renal impairment
4. Severe sepsis
5. Metabolic acidosis
6. Decompensated type 2 respiratory failure
7. Severe congestive cardiac failure
8. Acute coronary syndrome
9. Hepatic insufficiency
10. Excessive alcohol consumption
11. Malnourished or at high risk for malnutrition
12. Moribund or not for active treatment

13 Admitted to critical care unit

14. Unable to give informed consent
15. Pregnancy or lactation

Study drugs, Dose and Mode of Administration

Metformin hydrochloride 1000 mg twice daily or matched placebo, administered orally.

Duration of Treatment

28–35 days

Criteria for evaluation

Primary endpoint:

- Mean capillary glucose concentration during hospitalisation period.

Secondary endpoints:

- Clinical efficacy: COPD Assessment Test score, Exacerbation of Chronic Pulmonary Disease Tool (EXACT) score, time to medical fitness for discharge, time to actual discharge, rates of recurrent exacerbation, readmission (any cause), or death (all cause).
- Glycaemic control: mean daily insulin use during hospitalisation period, mean haemoglobin A1c and serum fructosamine concentrations at follow-up
- Inflammation and infection: mean C-reactive protein and serum cytokine concentration at discharge and follow-up (absolute values and change from baseline)
- Other metabolic effects: mean body mass index at follow-up, mean waist circumference at follow-up
- Airflow limitation: FEV_1 at 4 weeks
-

4 Introduction

4.1 Background

4.1.1 Chronic obstructive pulmonary disease (COPD)

Chronic obstructive pulmonary disease is the fourth leading cause of death worldwide, and a major cause of morbidity.[1] In the UK, it affects some 3 million people and causes more than 30,000 deaths per year.[2] COPD is usually caused by smoking, and is more common among individuals living in deprived areas.[3] Most people affected are over 65-years-old.[4] It is characterised by chronic inflammation and tissue injury in the lungs, and a range of effects outside the lungs, including skeletal muscle weakness, weight-loss, and cardiovascular disease.[5] For the patient, it causes breathlessness, cough, sputum production, and reduced quality of life.

4.1.2 COPD exacerbation

The natural history of COPD is a gradual, progressive deterioration, punctuated by episodes of acute worsening, termed exacerbations. In a cohort of patients with severe COPD, the median exacerbation rate was 3 per year.[6] Exacerbations often necessitate hospital admission—in 2004, COPD accounted for 111,077 emergency admissions to hospitals in the UK, and over one million hospital bed-days.[7] Health-related quality of life is substantially worse in patients who have frequent exacerbations.[6] Recovery from exacerbations is often slow,[8] and the effect of treatment is modest.[9] The 90-day mortality rate following hospitalisation for an exacerbation is 13.9%, and it is estimated that some 25% will have died within one year.[4] In a study of male patients with previously stable COPD, those who were hospitalised for COPD exacerbation had a the 5-year mortality rate of 55.2%.[10] By way of comparison, 5-year mortality rates for men with malignant melanoma, prostate cancer, and rectal cancer, were recently reported at 17.8%, 20.3% and 47.2%, respectively.[11] There is a clear need to develop new therapeutic strategies to improve management and outcomes of COPD exacerbations.

4.1.3 Stress hyperglycaemia

When an abnormally high concentration of glucose in the blood (hyperglycaemia) is provoked by an acute illness, it is termed stress hyperglycaemia. Stress hyperglycaemia is a common finding in hospitalised patients—including those without diabetes mellitus—and is associated with a range of detrimental pathophysiological effects.[12] Substantial observational data support a consistent relationship between blood glucose concentration and adverse outcomes in hospitalised patients across a range of conditions,[13] including COPD exacerbations. In a study of 348 patients hospitalised for COPD (5% of whom were diabetic), a blood glucose concentration >6.1 mmol/L was identified in 72%, and >11.1 mmol/L in 11%.[14] This was associated with an increased likelihood of death or prolonged hospital stay, the absolute risk of which increased by 15% (95% CI 4–27%) for every 1 mmol/L rise in blood glucose concentration.[14] Other studies have found hyperglycaemia to predict failure of non-invasive ventilation in the context of COPD exacerbations.[15,16] It is likely that hyperglycaemia in COPD exacerbations is caused by induction of peripheral insulin resistance, which may be caused by hypoxia,[17] acidosis,[18] systemic inflammation,[19] and inhaled[20] or systemic[21] corticosteroid treatment.

4.1.4 Management of hyperglycaemia in patients with critical illness

Whereas the association between hyperglycaemia and adverse outcomes is well-defined, it is not clear that the relationship is causal—such that lowering blood glucose may improve outcomes. Studies of tight glycaemic control with insulin in the critical care setting have yielded mixed results. In a single-centre, randomised controlled trial, it reduced mortality by 42% compared with standard treatment.[22] In a large multicentre trial, however, it was associated with a small excess risk of death.[23] Severe hypoglycaemia—an independent risk factor for death[24]—was observed in 6.8% of patients receiving intensive treatment versus 0.5% in controls (p<0.001). This serious adverse effect of insulin therapy may have contributed to the unfavourable outcome.

4.1.5 Management of hyperglycaemia in non-critically ill hospitalised patients

Hypergycaemia is associated with adverse outcomes in patients admitted with acute coronary syndromes.[25] In the DIGAMI trial[26] of insulin–glucose treatment in myocardial infarction, a lower mean blood glucose in the intervention group was associated with a 29% reduction in 1-year mortality (p=0.027). This was not reproduced in the subsequent DIGAMI 2[27] and CREATE-ECLA[28] trials, but in these, the mean blood glucose concentration was no lower in the intervention group than in controls. Based on available evidence, guidelines from the Scottish Intercollegiate Guidelines Network,[29] the American Heart Association,[30] and European Society of Cardiology[31,32] have recommended glucose-lowering treatment in diabetic and hyperglycaemic patients with acute coronary syndromes.

No prospective, controlled trial data exist to guide the management of hyperglycaemia in other non-critical illnesses, and current guidelines are therefore based on expert recommendations.[33] They recommend that most non-critically ill hospitalised patients treated with insulin should have a premeal glucose <7.8 mmol/L and random blood glucose concentrations <10.0 mmol/l, provided these targets can be safely achieved.

4.2 Investigational Medicinal Product (IMP)

Metformin hydrochloride (*N,N*-dimethylimidodicarbonimidic diamide hydrochloride) is an orally-administered drug that is chemically and pharmacologically distinct to all other classes of anti-hyperglycaemic drugs. It is a first-line treatment for most patients with type 2 diabetes mellitus who are not adequately controlled by dietary and lifestyle means alone.[34] Its predominant action is to lower blood glucose by reducing hepatic glucose output and improving peripheral insulin sensitivity.[35] Uniquely amongst oral anti-diabetic drugs, metformin does not produce hypoglycemia either in patients with type 2 diabetes or normal subjects, unless used in combination with other antidiabetic agents.[36]

Unlike insulin, which is complex to administer and associated with a substantial risk of hypoglycaemia,[23,24] metformin is a simple oral treatment which offers the potential to improve glycaemic control without an attendant risk of hypoglycaemia. However, apart from small studies in patients with severe burn injuries,[37,38] whether this holds true in stress hyperglycaemia has not previously been studied. Patients with COPD represent a rational cohort

in which to study this effect, as the prevalence of hyperglycaemia is high, and its association with adverse outcomes has been characterised.[14,15,16]

The main side effect of metformin is gastrointestinal upset, notably diarrhoea, and this is usually transient.[39] While metformin is generally considered a safe drug,[35] it is rarely linked with lactic acidosis, a serious condition associated with high mortality in the absence of prompt treatment. However, the evidence for this, in the absence of an elevated metformin concentration due to overdose or renal failure, is sparse. Between 1995 and 2000, 22 cases meeting the accepted definition were reported in the medical literature.[40] Metformin was believed to be the precipitant in 12 cases, 11 of whom had metformin accumulation due to renal failure. There are no clinical trial data to support an association between metformin therapy and lactic acidosis. In a Cochrane review of 347 trials involving metformin therapy,[41] no cases of lactic acidosis were identified in 70,490 patient-years of exposure. The upper limit for the true incidence of lactic acidosis per 100,000 patient-years was estimated at 4.3 cases in the metformin group and 5.4 cases in the non-metformin group.

4.3 Metformin—pre-clinical data

4.3.1 Pharmacokinetics

In clinical practice, metformin is administered orally. It has a bioavailability of 50–60%, and reaches a maximal plasma concentration of 1–2 μg/mL, 1 to 2 hours after an oral dose of 500–1000 mg.[35] It undergoes negligible plasma protein binding and is distributed to most tissues in concentrations comparable to those in plasma.[35,36] Steady-state plasma concentrations are reached within 24–48 hours and are usually <1 μg/mL.[36] Its plasma elimination half-life is 1.5–6.2 hours; its blood elimination half-life is approximately 17.6 hours, suggesting that it partitions into erythrocytes.[35,36] It is excreted unchanged in the urine by means of tubular secretion and glomerular filration.[36] No metabolites have been identified in humans.[36]

4.3.2 Mechanism of action

The glucose-lowering effect of metformin is understood to arise through reduced hepatic glucose output and enhanced peripheral glucose uptake.[42] In hepatocytes, metformin suppresses gluconeogenesis.[43] Peripherally, in muscle, it increases insulin-stimulated glucose uptake, leading to increased glucose oxidation and glycogen production.[44] To a lesser extent, in adipose tissue, it stimulates uptake and oxidation of glucose and lipogenesis.[35] Metformin promotes weight loss in obese patients with type 2 diabetes mellitus; this is largely confined to the loss of body fat rather than lean body mass.[42]

The molecular target of metformin is the kinase LKB1, which regulates the activity of the enzyme AMP-activated protein kinase.[45] AMPK has been described as a 'master-switch in the control of whole-body energy and substrate metabolism'.[46] Its activation inhibits enzymes involved in gluconeogenesis and glycogen synthesis in hepatocytes, and stimulates insulin signalling and glucose transport in muscle cells.[46] This is understood to underlie the effects of metformin on glucose and lipid metabolism.[47]

4.3.3 Anti-inflammatory and anti-oxidant effects

Metformin appears to have actions beyond its effects the metabolic pathways described above. In human vascular smooth muscle cells, macrophages, and endothelial cells, metformin (at therapeutically-relevant concentrations) has a dose-dependant inhibitory effect on the release of pro-inflammatory cytokines IL-6 and IL-8.[48] This appears to be mediated by diminished activation and nuclear translocation of the proinflammatory transcription factor NF-κB (nuclear factor-kappa B).[48] Likewise, in human umbilical vein endothelial cells, TNFα-induced NF-κB activity and IL-6 production is inhibited by metformin.[49]

There is also evidence to suggest that metformin has favourable effects on oxidative stress–antioxidant balance. In high fructose-fed rats, metformin administration appears to have an antioxidant activity independent of its effect on insulin activity.[50] *In vitro* experiments have shown that metformin is able to scavenge hydroxyl (•OH) free radicals.[51] Clinical studies suggest that these effects also exist *in vivo* when metformin is administered to diabetic patients.[52]

4.4 Metformin—clinical data

4.4.1 Effects and uses in type 2 diabetes mellitus

The anti-hyperglycaemic action of metformin is supported by substantial clinical trial data. In a meta-analysis of 9 randomised controlled trials, the weighted mean difference between metformin and placebo for fasting blood glucose was -2.0 mmol/L (95% CI -2.4 to -1.7), and -0.9% (95% CI -1.1 to -0.7) for haemoglobin A1c (HbA1c).[53] In a Cochrane review, metformin was found to reduce fasting plasma glucose and HbA1c, and improve weight, lipid profile, hyperinsulinaemia, and diastolic blood pressure.[54]

In patients with type 2 diabetes, metformin reduces the risk of death and other diabetes-related endpoints,[55] and is recommended for first-line treatment in most patients.[34] In pre-diabetic patients, compared with placebo, it reduces the risk of progression to diabetes.[56]

It is notable that in the influential UK Prospective Diabetes Study,[55] reductions in mortality and other diabetes-related endpoints were observed only among patients randomised to metformin. This was despite similar levels of glycaemic control in patients treated with a sulphonylurea or insulin. This has led to speculation that metformin may have vasoprotective properties beyond to its glucose-lowering effect.[57,58]

4.4.2 Anti-hyperglyaemic effects in acute illness

Studies on the effects of metformin in stress hyperglycaemia are sparse, and limited to small trials in patients who had suffered severe burns injuries. In one, 10 non-diabetic patients with severe burn injuries were randomised equally, and in a double-blinded fashion, to metformin or placebo.[37] At 7 days, patients treated with metformin had a significantly lower mean plasma glucose than those receiving placebo (7.4 *vs* 10.8 mmol/L, $P<0.05$), and higher rates of glucose clearance.

In a second study, 16 non-diabetic patients with severe burn injuries were randomised, in a double-blinded fashion, to metformin or placebo.[38] The mean plasma glucose during the 7-day study period was lower among patients treated with metformin than in those who received placebo (7.7 *vs* 10.2 mmol/L respectively, $P<0.05$), as was their mean insulin requirement (14 *vs*

4 units per day, P<0.05). Evidence was also found that metformin may reduce muscle protein catabolism in this setting.

4.5 Potential benefits of metformin therapy in the context of COPD exacerbation

4.5.1 Anti-hyperglycaemic action

The 5-year mortality rate following hospitalisation for COPD is worse than that of many cancers. Observational data suggest that, for each 1 mmol/L increase in blood glucose concentration in the context of COPD exacerbation, there is a 15% increase in the absolute risk of death or prolonged hospitalisation.[14] It would therefore seem important that anti-hyperglycaemic treatment is explored as a potential therapeutic avenue. However, glycaemic control with insulin is fraught with complexities. It is associated with a risk of hypoglycaemia—a common and dangerous adverse effect.[23,24] By virtue of its narrow therapeutic range, it requires close monitoring and frequent dose-adjustment. Metformin, by contrast, is simple to administer and has a broader therapeutic range. As a result of its action to lower blood glucose concentration towards, but not below, the normal range, it may be administered while the blood glucose concentration is normal without risk of hypoglycaemia. Thus it may be used in a pre-emptive, rather than reactive, anti-hyperglycaemic strategy. This may be particularly advantageous in the context of COPD exacerbations, when hyperglycaemia may not be evident initially, but may develop after systemic corticosteroids are introduced. Likewise, metformin may be continued during the outpatient recovery phase, without need for dose-adjustment after the cessation of systemic corticosteroids. These observations lead us to speculate that it may be a valuable agent for the treatment and prevention of hyperglycaemia in the context of COPD exacerbation. We further speculate that this may lead to meaningful clinical benefit.

4.5.2 Reduced requirement for insulin

In stress hyperglycaemia associated with severe burn injuries, metformin was found to reduce the need for insulin treatment.[38] This finding is consistent with the drug's known pharmacological effects. As insulin is a potentially dangerous drug, with a narrow therapeutic range, this may be advantageous.

4.5.3 Favourable changes in oxidative stress–antioxidant balance

Metformin has favourable effects on oxidative stress–antioxidant balance in patients with diabetes mellitus.[52] Oxidative stress and reduced endogenous antioxidant capacity play an important role both provoking and sustaining chronic inflammation in COPD.[59,60,61] Endogenous antioxidant capacity is particularly reduced during COPD exacerbations, remaining low for several days, and returning to normal at the time of clinical recovery.[62] These findings have led to the suggestion that antioxidant compounds may represent important novel treatments for COPD, and calls for their evaluation in clinical trials.[60] The antioxidant effects of metformin in the context of COPD exacerbation, and its effect on clinical outcomes, have not previously been characterised.

4.5.4 Improving steroid sensitivity

Corticosteroids are potent anti-inflammatory agents, which are highly effective in the treatment of many inflammatory and immune diseases. However, their efficacy in COPD has been called into question, based on a hypothesis that the inflammatory phenotype in COPD is steroid-resistant.[63] The putative driver for steroid resistance in COPD is oxidative stress,[63] and this has further strengthened the case for the evaluation of antioxidant therapies in this setting. Metformin, with its antioxidant effects, represents a rational therapeutic strategy to target steroid resistance and thus potentially to improve their efficacy.

4.6 Assessment and management of risks

4.6.1 Minor side effects

The side effects of metformin are predominantly gastrointestinal. In a meta-analysis comparing metformin with placebo,[54] the risk ratio for diarrhoea was 3.09 (1.58–6.07). The rate of gastrointestinal discomfort did not differ significantly between the groups. Diarrhoea associated with metformin is usually transient.[39]

The relevant summary of product characteristics (SmPC)[36] lists gastrointestinal disorders, such as nausea, vomiting, diarrhoea, abdominal pain and loss of appetite, as very common (frequency >1/10). It states that these side effects 'occur most frequently during initiation of therapy and resolve spontaneously in most cases.' Taste disturbance is listed as a common side effect (frequency >1/100). All other side effects are described as very rare (<1/10,000) or not known (cannot be estimated from available data).

The protocol includes provisions to deal with the occurrence of minor side effects, which may include dose-reduction, suspension, or discontinuation of the study drug as appropriate. In accordance with the SmPC,[36] to minimise gastrointestinal effects as far as possible, the study tablets will be administered after meals, and the dose will be escalated gradually to the target dose over 4 days.

4.6.2 Hypoglycaemia

Metformin does not induce hypoglycaemia when used as the sole glucose-lowering therapy, and does not exert a hypoglycaemic action in non-diabetic patients unless given in overdose.[36,39] Even in the setting of overdose, hypoglycaemia has not been seen with metformin doses up to 85 g (more than the total dose used over the duration of this study).[36]

The use of other oral glucose-lowering agents will not be permitted in this trial. The use of insulin will be permitted for patients with persistent, significant hyperglycaemia, occurring during the inpatient phase, at the treating physicians' discretion. The risk of hypoglycaemia attendant with insulin treatment would exist irrespective of whether the patient was enrolled in this trial or taking metformin. No additional glucose-lowering therapy will be permitted during the outpatient phase.

Although not listed in the SmPC, it is suggested that there may be a risk of hypoglycaemia if metformin is administered in the setting of malnutrition, inadequate caloric intake, or excessive alcohol consumption. For this reason, the nutritional status of all patients will be assessed on study entry. Those considered to be at high risk of malnutrition shall be excluded. Those at

moderate risk will be subject to increased nutritional monitoring. Any patient in whom nutrition is withheld (for example, for procedures requiring the patient to be fasted) or inadequate, shall have their study treatment suspended until adequate nutrition is resumed. Likewise, potential participants with excessive alcohol consumption will be excluded. Any individual who develops symptoms or signs of alcohol withdrawal (and/or requires treatment for this) will have the study treatment discontinued.

The blood glucose concentration will be monitored regularly and frequently in all patients during the inpatient phase. Any patient who develops severe hypoglycaemia in the absence of concomitant therapy with another glucose-lowering agent shall have the study treatment discontinued.

4.6.3 Lactic acidosis

Lactic acidosis is a rare but serious condition, with a mortality rate between 8% and 50%.[41] It is characterised by the accumulation of lactic acid in the blood (>5.0 mmol/L), decreased blood pH (<7.35), and a high anion gap. It occurs most commonly in conditions associated with impaired tissue oxygenation (type A lactic acidosis). Where there is no evidence of impaired tissue oxygenation, it is termed type B lactic acidosis.

Treatment with another biguanide, phenformin, was associated with lactic acidosis. For this reason, concern was raised that metformin may be associated with the same condition. However, metformin differs from phenformin in several important respects. Whereas phenformin requires hepatic metabolism, metformin is excreted unchanged in urine. Phenformin demonstrably increases plasma lactate concentration, inhibits lactate oxidation, impairs oxidative phosphorylation, and increases lactate release from muscle.[42] Metformin, by contrast, reduces lactate utilisation for gluconeogenesis, but increases lactate oxidation, resulting in no net change in plasma lactate concentration.[42] *In vitro,* both phenformin and metformin impair mitochondrial function leading to ATP depletion and lactate efflux in a dose-dependent manner, but their potencies in this respect differ approximately 150-fold.[64]

In a Cochrane review of 347 trials,[41] no cases of lactic acidosis were identified in 70,490 patient-years of metformin exposure. The upper limit for the true incidence of lactic acidosis per 100,000 patient-years was estimated at 4.3 cases in the metformin group and 5.4 cases in the non-metformin group. Compared with placebo and non-biguanide therapies, the initiation of metformin was not associated with a net change in the mean lactate level. During treatment, lactate levels did not differ significantly between the metformin and non-metformin groups. The mean lactate level was lower in the metformin group than it was in the phenformin group (absolute difference 0.75 mmol/L, 95% CI -0.85 to -0.65). Although it was not possible to quantify the number of patients included in the trials who had contraindications to metformin use, it was noted that 324 (97%) trials in the analysis allowed for the inclusion of subjects with at least one contraindication.

Thus the putative association between metformin therapy and lactic acidosis is based largely on case reports. These suggest that there is a risk of lactic acidosis when the plasma concentration of metformin is allowed to accumulate.[36] This is invariably a consequence of renal failure or overdose. Between 1995 and 2000, 22 cases were identified in a review of the medical

literature.[40] Among the 12 cases in which metformin was considered the likely cause of lactate accumulation, all but one patient had renal failure. One patient (8%) died in this group, and this was attributed to the patient having declined dialysis therapy. By contrast, death occurred in 6/8 patients (75%) whose lactic acidosis was felt to have been due to another pathology. Another series reported 42 cases of metformin-associated lactic acidosis presenting over a 10-year period. All had supra-therapeutic plasma metformin concentrations, 13 due to deliberate overdose and 29 due to incidental overdose.

The safety of metformin in the context of co-morbidities was examined in a prospective interventional study by Rachmani *et al.*[65] These investigators identified 393 patient admitted to hospital, who were taking metformin despite at least one traditional contraindication to its use. The cohort included 91 patients with severe COPD (forced expiratory volume in 1 second ≤50% predicted). Participants were randomised to either continue or stop metformin therapy, and followed for four years. No cases of lactic acidosis occurred in either group, and plasma lactate concentrations did not differ.

Renal impairment and advanced liver disease are consistently regarded as contraindications to metformin therapy. Pulmonary disease, due to its associated risk of tissue hypoxia, is regarded as a contraindication by some,[66] but is widely disregarded in clinical practice.[66,67] An audit conducted in this hospital identified that among a cohort of 108 patients with diabetes mellitus who were admitted with a COPD exacerbation, metformin was listed as part of the admission drug history for 38 (35%). Among those patients who were already prescribed metformin, it was continued during the admission in 25 (76%).

In this study, we consider there to be a risk of lactic acidosis related to the underlying condition (COPD) and commonly associated comorbidities (such as respiratory failure, heart failure, renal failure and severe sepsis). As noted above, the lactate levels and prognosis observed in cases of lactic acidosis are believed to be dictated by with the severity of the underlying condition, rather than to the presence or plasma concentration of metformin.[40,68] Nevertheless, given the lack of certainty over the safety of metformin therapy in such settings, and to avoid potential confusion over the aetiological agent, we shall stop or suspend the study drug if a condition develops in which there is a high risk of lactic acidosis, or if the plasma lactate concentration rises above pre-specified levels.

Further, we consider that there is a small but genuine risk of metformin-associated lactic acidosis if metformin is allowed to accumulate due to renal failure. To mitigate this, we will monitor the renal function regularly while the participant is an inpatient (when, due to the severity of their concurrent illnesses, the risk of developing renal failure is greatest), and stop or suspend the study treatment if the estimated GFR falls, or the serum creatinine rises, above pre-specified levels.

We judge that the risk of metformin-associated lactic acidosis outside the situations described above is extremely low. This risk must be balanced against the seriousness of the condition under study. As noted above, the 90-day mortality rate following hospitalisation for a COPD exacerbation is 13.9%; the 5-year mortality rate exceeds 50%. Observational data suggest that

lowering blood glucose concentration may offer the prospect of substantial benefit. As such, our assessment is that the risk–benefit balance lies in favour of undertaking this trial.

5 Study objectives

The overarching research question for this body of work is to ascertain whether metformin improves recovery from COPD exacerbations. This pilot study will address two more limited questions, as a basis for future trial design.

5.1 Primary objective

To determine whether, in patients hospitalised for COPD exacerbations and receiving conventional treatment, the addition of metformin, as compared with placebo, ameliorates hyperglycaemia.

5.2 Secondary objectives

To determine whether, in patients hospitalised for COPD exacerbations and receiving conventional treatment, the addition of metformin, as compared with placebo, produces effects on clinical outcome, and measures of inflammation, steroid sensitivity, and airflow obstruction, which merit further study.

The data collected in relation to the secondary objectives will be hypothesis-generating. They will also be used to inform the selection of appropriate end points, and as a basis for sample size calculations, for a future larger study.

6 Trial design

6.1 Overall design

This is a randomised, double-blind, parallel-group, placebo-controlled trial. An overview of the study is provided in the accompanying schematic diagram.

A double-blinded design has been adopted to provide the best possible evidence for the efficacy of metformin in this context by minimising the risk of bias. Blinding will be implemented by means of visually identical active and placebo treatments.

6.2 Study population

The sample size was originally set at 46 patients, randomised equally to metformin or placebo. From Substantial Amendment 4 approval, this is increased to 69 patients, randomised to metformin or placebo in a 2:1 ratio. The patients will be drawn from the population of patients admitted to the study sites with an exacerbation of COPD, and who are able to enter the study within 48 hours of admission.

6.3 Dosage regimen and rationale

The dose range for metformin hydrochloride is 500–2550 mg/day. However, the maximal anti-hyperglycaemic effect is achieved at 2000 mg/day[46] and this is the usual maximal dose.[39] To minimise the risk that a negative result arises as a consequence of an insufficient dose, we have selected a dose of 2000 mg/day.

It is recommended that metformin should be given in divided doses with or after meals.[36] Metformin 2000 mg/day can be conveniently administered as two 500 mg tablets taken twice daily. Ideally, doses should be equally separated, but for practical purposes we will recommend that they be taken at least 8 hours apart, with or up to 20 minutes after breakfast and evening meals. This recommendation is broadly consistent with that used in a large randomised controlled trial.[69]

6.4 Treatment period

The duration of hospitalisation for COPD exacerbations is variable. In a study conducted in this hospital,[14] the median length of stay was 9 days (interquartile range, 5–17 days). In a more recent audit and service evaluation study in this hospital, of patients with diabetes and COPD exacerbations, the median length of stay was 8 days (interquartile range, 4–16 days; range 0–75 days). To avoid the introduction of bias (whereby, for example, individuals who were less unwell and thus had a shorter stay would have a lower exposure to the study drug), we have elected to adopt a standardised treatment period for all participants. A period of 28–35 days was selected, to capture the full duration of hospitalisation for the considerable majority of patients, and most of the recovery period. In a prospective study of 91 patients, who experienced 504 exacerbations, the total symptoms score had recovered by day 35 in 86.1% of exacerbations. A 7-day period of leeway was selected to take account of potential difficulties arranging a follow-up appointment (due to weekends, public holidays, and patient/staff availability), while ensuring that the study drug can be continued until the date of that appointment. Follow-up appointments will take place at the site of recruitment.

6.5 Concomitant treatment

All participants will receive systemic corticosteroids (prednisolone 30 mg daily) for a minimum of 7 days. Otherwise, all care for the COPD exacerbation and co-morbidities will be at the discretion of the treating physicians. Tapering and cessation of steroid therapy and any use of antibiotics will also be at the discretion of the treating physician.

The use of other oral anti-hyperglycaemic medications will not be permitted. However, the use of insulin will be permitted during the inpatient phase for the treatment of hyperglycaemia that is significantly and persistent above 7.8 mmol/L (pre-meal readings) and/or 10 mmol/L (random readings), in line with current guidelines.[33] The decision to administer insulin under these circumstances, and the choice formulation and dose, will be at the discretion of the treating physician.

6.6 Schematic of trial design

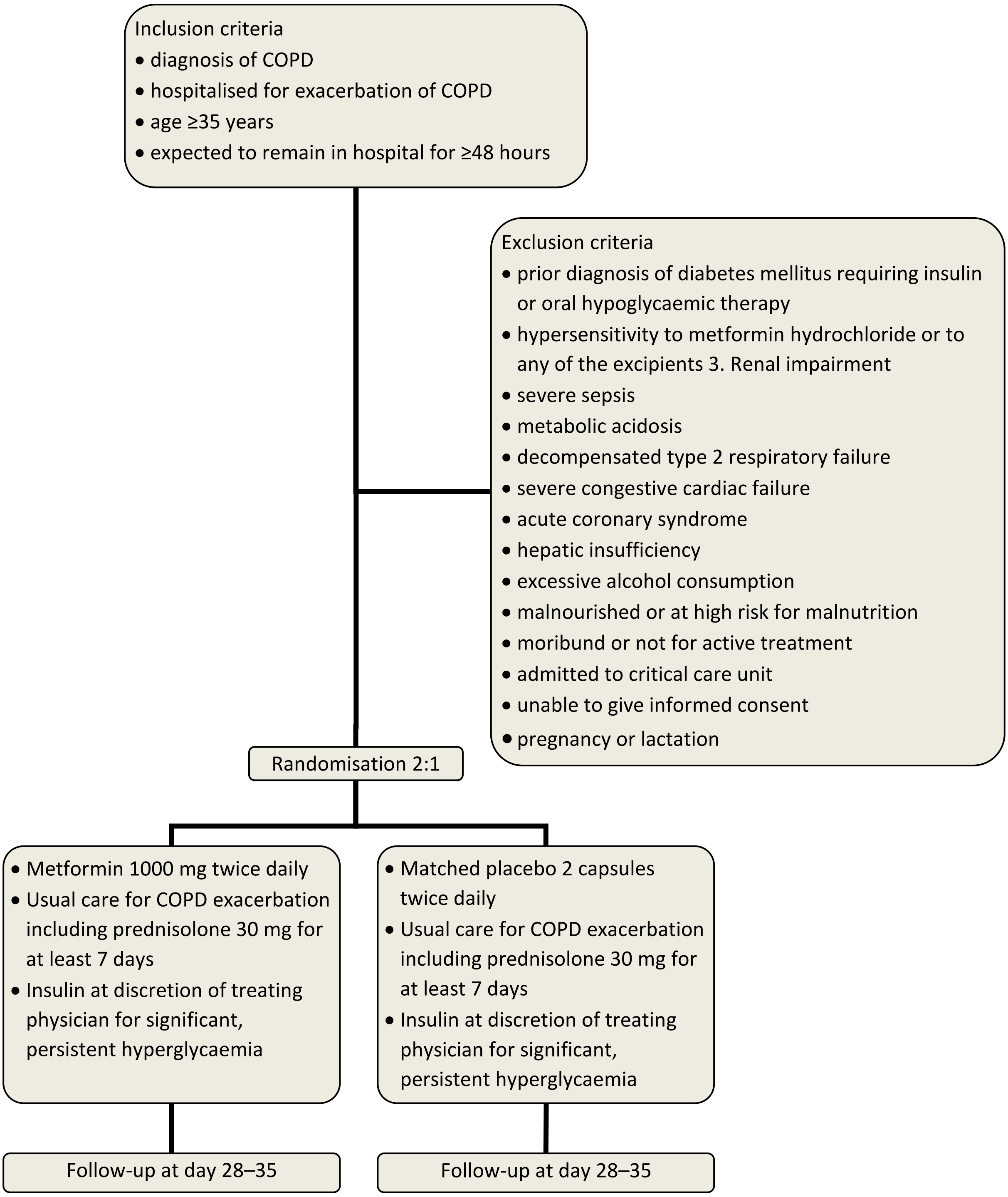
Inclusion criteria
• diagnosis of COPD
• hospitalised for exacerbation of COPD
• age ≥35 years
• expected to remain in hospital for ≥48 hours
Exclusion criteria
• prior diagnosis of diabetes mellitus requiring insulin or oral hypoglycaemic therapy
• hypersensitivity to metformin hydrochloride or to any of the excipients 3. Renal impairment
• severe sepsis
• metabolic acidosis
• decompensated type 2 respiratory failure
• severe congestive cardiac failure
• acute coronary syndrome
• hepatic insufficiency
• excessive alcohol consumption
• malnourished or at high risk for malnutrition
• moribund or not for active treatment
• admitted to critical care unit
• unable to give informed consent
• pregnancy or lactation
Randomisation 2:1
• Metformin 1000 mg twice daily
• Usual care for COPD exacerbation including prednisolone 30 mg for at least 7 days
• Insulin at discretion of treating physician for significant, persistent hyperglycaemia
• Matched placebo 2 capsules twice daily
• Usual care for COPD exacerbation including prednisolone 30 mg for at least 7 days
• Insulin at discretion of treating physician for significant, persistent hyperglycaemia
Follow-up at day 28–35
Follow-up at day 28–35

7 Inclusion and exclusion criteria

7.1 Inclusion criteria

(1) Diagnosis of COPD based on at least one of the following criteria:

- Previously recorded airflow obstruction on spirometry (FEV1 <80% predicted, FEV1:FVC <70% predicted), without evidence of complete reversibility
- Previous diagnosis of COPD by a respiratory physician
- Exertional breathlessness, chronic cough, or regular sputum production, in a patient who has ≥10 pack-years smoking history

(2) Hospitalisation for exacerbation of COPD, defined by the presence of at least two of the following major features, or one major symptom and one minor feature.

Major features:

- Increased breathlessness
- Increased sputum volume
- Increased sputum purulence

Minor features:

- upper respiratory infection within the past 5 days
- fever without other cause
- increased wheezing or cough
- increase in respiratory rate or heart rate by 20% as compared with baseline

(3) Age ≥35 years

(4) Expected to remain in hospital for ≥48 hours.

7.2 Exclusion criteria

Any one of the following features

**Criterion**	**Definition**
Prior diagnosis of diabetes mellitus requiring insulin or oral hypoglycaemic therapy	Patient-reported diagnosis of diabetes, or a documented record of diabetes mellitus in the available clinical notes, requiring insulin or oral hypoglycaemic treatment
Hypersensitivity to metformin hydrochloride or to any of the excipients	Patient-reported prior reaction, or documented in available case notes
Renal impairment	Estimated glomerular filtration rate (eGFR) <45 mL/minute/1.73 m^2 (using the modification of diet in renal disease (MDRD) four-variable equation), serum creatinine concentration >130 µmol/L, or a requirement for renal-replacement therapy

Severe sepsis	Sepsis with at least one feature of organ dysfunction or tissue hypoperfusion.<br><br>**Sepsis** is defined as suspected or proven infection, plus **at least two** of the following features (unless they are normal for that patient at baseline):<br>• temperature >38.3 or <36°C<br>• white cell count <4 or >12 × 10^9/L<br>• acutely altered mental status<br>• blood glucose >8.3 mmol/L<br><br>**Severe sepsis** is defined as sepsis in conjunction with **at least one** feature of organ dysfunction or tissue hypoperfusion:<br>• renal: creatinine >177 µmol/l or UO <0.5 ml/kg/hr for 2 hrs<br>• hepatic: bilirubin >34 µmol/L<br>• coagulation: platelets <100 x 10^9/L, INR>1.5 or APTT >60 seconds<br>• tissue hypoperfusion: any one of, systolic blood pressure systolic <90 mmHg, mean blood pressure <65 mmHg, a reduction of >40 mmHg from the patient's normal systolic blood pressure, or lactate >2 mmol/L.
Metabolic acidosis	Arterial or venous blood pH below the lower limit of normal and an arterial or venous bicarbonate concentration (measured or calculated) below the lower limit of normal
Decompensated type 2 respiratory failure	Partial pressure of carbon dioxide in arterial blood higher than the upper limit of normal, with arterial blood pH below lower limit of normal
Severe congestive cardiac failure	Clinical diagnosis of congestive cardiac failure by the treating physicians on the basis of typical symptoms and/or signs and/or results of investigations, which is felt to be the cause of shortness of breath at rest
Acute coronary syndrome	Clinical diagnosis of acute coronary syndrome by the treating physicians on the basis of typical symptoms and/or signs and/or results of investigations, and for which the patient is receiving treatment (likely to include aspirin, clopidogrel and low molecular weight heparin or fondaparinux, depending on local policy) at the time of screening
Hepatic insufficiency	Previous clinical, radiographic or histological diagnosis of liver cirrhosis, as reported by the patient or recorded in their hospital case notes; or a clinical suspicion of liver disease in conjunction with a serum bilirubin concentration >34 µmol/L
Excessive alcohol consumption	Patient-reported regular alcohol consumption of ≥56 units per week for men or ≥42 units per week for women, representing more than twice the UK recommended upper limits for sensible drinking
Malnourished or at high risk for malnutrition	Malnutrition Universal Screening Tool (MUST)[70] score ≥2
Moribund or not for active treatment	Treating clinician and/or investigator judgement that the patient is unlikely to survive to hospital discharge, and/or the resulting discontinuation of treatments for the underlying condition
Admitted to critical care unit	Current admission of the patient to the any intensive care or high dependency unit, or a plan to do so imminently
Unable to give informed consent	An investigator or physician judgement that the patient lacks the capacity to reach an informed decision on the appropriateness of their participation in the trial, due to an inability to:<br>• understand the information relevant to the decision<br>• retain that information<br>• use or weigh that information as part of the process of making the decision, or<br>• communicate that decision
Pregnancy or lactation	Patient-reported pregnancy or lactation, and/or a positive result from a urinary pregnancy test

8 Recruitment

Patient recruitment at a site will only commence once the trial team has ensured that the following approval/documents are in place:

- The REC and regulatory (MHRA) approvals
- A signed Clinical Trial Site Agreement (CTSA)
- Final sponsorship and host site approval
- The site/trial initiation visit has taken place

8.1 Subject recruitment process

Potential participants will be identified by doctors in the clinical care team, who are also part of the study team. Potentially eligible participants will be identified as those likely to meet the inclusion criteria. Potential participants shall be approached by a doctor from the clinical care and study teams, and informed them that a research project is underway, which they may be eligible to take part in should they wish. If the patient requests further information, the doctor will provide an overview of the project, including its purpose, nature, burdens, and risks; and will emphasise that participation is entirely voluntary. If the patient expresses interest in participating in the trial, the doctor will provide them with a copy of the patient information leaflet and explain its contents. If the patient remains positive about potential participation, informed consent will then be sought.

9 Study procedures and schedule of assessments

9.1 Informed consent

Informed consent will be obtained by the Principal Investigator, Chief Investigator, or a nominated deputy as recorded on the delegation log. Only those members of the study team who have clinical responsibility for the care of patients under the care of the general medical service will be permitted to undertake informed consent. All individuals taking informed consent will have received training in Good Clinical Practice and the study protocol, and have been delegated this duty by the CI/PI on the delegation log.

Consent to enter this study will be obtained after a full account has been provided of its nature, purpose, risks, burdens and potential benefits, and the patient has had the opportunity to deliberate. The patient will be allowed to specify the time they wish to spend deliberating, usually up to 24 hours. Periods shorter than 24 hours will be permitted if the patient feels that further deliberation will not lead to a change in their decision, and provided the person seeking consent is satisfied that the patient has deliberated sufficiently on the information given. This provision has been made with the support of our Patient Advisory Group. Likewise, periods longer than 24 hours will be permitted should the patient request this and while they remain potentially eligible for study entry.

The Investigator or designee will explain that the patients are under no obligation to enter the trial and that they can withdraw at any time during the trial, without having to give a reason.

A copy of the signed Informed Consent will be given to the participant. The original signed form will be retained at the study site.

If new safety information results in significant changes in the risk–benefit assessment, the patient information leaflet (PIL) will be reviewed and updated if necessary. All subjects, including those already being treated, will be informed of the new information, given a copy of the revised PIL and their consent to continue in the study will be sought.

9.2 Randomisation procedure

Prior to Substantial Amendment 4 approval, patients were allocated to metformin or matched placebo by equal group random allocation in blocks of four at Chelsea and Westminster Hospital and blocks of six at St George's Hospital and other sites. Following Substantial Amendment 4 approval, patients will be allocated in a 2:1 ratio to metformin or placebo in blocks of six, with a corresponding increase in the sample size. This amendment was made to increase the number of patients exposed to active drug, so that more robust conclusions can be drawn with respect to the feasibility, safety and tolerability of this agent in the trial population.

Randomisation lists will be produced in advance by the drug manufacturer (Bilcare [GCS] Europe Ltd), and will be provided to the Pharmacy department for the purposes of allocation and/or emergency unblinding. An SOP on the blinding and unblinding procedure will be prepared prior

to study commencement. Randomisation will be performed by the IMP manufacturer in accordance with the method agreed by the study team and trial statistician.

Following allocation to a study treatment, patients will be given a trial participant card, which will have the study title, IMP details, patient trial number and contact details for advice and emergency unblinding.

9.3 Emergency unblinding

The blinded treatment allocation will be held within the Pharmacy Site File. Access to the randomisation code for a participant before the completion of the trial will be permitted in exceptional circumstances if it is necessary to determine subsequent best care for the participant, or for another person suspected to have ingested the study treatment of a known trial participant.

In the event of an emergency and/or clinical requirement for the blinded study treatment allocation to be revealed, the enquirer will contact the relevant Pharmacy Department as described on the participant study card. This service is available 24 hours per day. During working hours, the enquirer should contact the Pharmacy Clinical Trials team. Outside of office hours, the enquirer should request that switchboard bleep the on-call pharmacist. The Pharmacy Clinical Trials team procedures require that the Chief Investigator or Principal Investigator is contacted to obtain permission before treatment allocation is revealed to the enquirer.

9.4 Screening assessments

Patients' eligibility for inclusion shall be screened by reviewing the clinical history, concomitant medications, and examination, which all have already been obtained as part of routine care. Any components of the history or examination that are required to determine eligibility but have not been documented as part of routine care will be obtained during this visit.

9.5 Treatment procedure

Dosing schedule (treatment and placebo arms):

- Day 1: one capsule taken after food following first mealtime after randomisation
- Day 2: one capsule taken after breakfast time and one capsule after evening mealtime
- Day 3: two capsules taken after breakfast time and one capsule after evening mealtime
- Day 4 until follow-up (day 28–35): two capsules taken after breakfast time and two capsules after evening mealtime

9.6 Summary flow chart of study assessments

Day	IMP administration	Assessments
1	One capsule	Capillary glucose concentration (thereafter repeated before and 2 hours after each mealtime, and at approximately 22:00)<br>COPD Assessment Test (CAT)<br>Exacerbations of Chronic Pulmonary Disease Tool (EXACT)<br>Malnutrition Universal Screening Tool (MUST)<br>Renal profile, liver profile, CRP, lactate, random glucose, HbA1c<br>Serum sample saved
2	One capsule twice daily	Exacerbations of Chronic Pulmonary Disease Tool (EXACT)<br>Capillary glucose (as above)<br>CRP<br>Food chart if admission MUST score 1 (medium risk)
3	Two capsules morning, one capsule evening	Exacerbations of Chronic Pulmonary Disease Tool (EXACT)<br>Capillary glucose (as above)<br>CRP measured daily to day 7<br>Food chart if admission MUST score 1 (medium risk)
Day 4 until discharge	Two capsules twice daily	Exacerbations of Chronic Pulmonary Disease Tool (EXACT)<br>Capillary glucose (as above)<br>CRP measured daily to day 7<br>Safety parameters (renal function, liver enzymes, plasma lactate, venous glucose) measured at least every 72 hours until day 7 (more often if deemed necessary by study team or clinical team), then at a frequency appropriate for the clinical condition of the patient (no less often than once-weekly)
Day of discharge	Two capsules twice daily	Capillary glucose (as above, until time of discharge)<br>Exacerbations of Chronic Pulmonary Disease Tool (EXACT)<br>COPD Assessment Test<br>Spirometry<br>CRP<br>Serum sample saved
Day of discharge until follow-up	Two capsules twice daily	Exacerbations of Chronic Pulmonary Disease Tool (EXACT)
Follow-up (day 28–35)	Two capsules in the morning	Structured interview and pill count<br>COPD Assessment Test (CAT)<br>Weight, height and waist circumference<br>Spirometry<br>Venous glucose, HbA1c, lactate, CRP<br>Serum sample saved
12 weeks	None	Vital status (electronic patient record)<br>Hospital admissions (self-reported and electronic patient record)<br>COPD Assessment Test (returned by post)<br>Antibiotic and steroid courses (self-reported)<br>Diagnosis and/or treatment for diabetes (self-reported)

9.7 Procedure for missed and duplicated study assessments

Where, for whatever reason, assessments are not performed at their scheduled time, they shall be performed as soon as possible after identification of the omission. For repeated measurements, if the delay is more than 50% of the interval between repeated measurements, then the measurement will be regarded as missed.

With respect to capillary blood glucose concentration measurements, missed measurements will be regarded as a protocol deviation if three or more measurements are missed in one calendar day, or if the interval between any two consecutive measurements exceeds 12 hours.

The study treatment will not be stopped or suspended for missed study measurements unless the Investigator determines that this poses a specific risk to the safety of the participant. This determination will be informed by the clinical condition of the patient, and results of prior measurements.

Where study assessments have been duplicated (for example, when additional measurements have been taken as part of clinical care), the first recorded measurement after the scheduled time for that assessment will be taken as the study assessment.

9.8 Methods

All blood samples will be taken using standard peripheral venepuncture technique. Measurement of height, weight, and waist circumference will be undertaken according to published guidelines.[71] The COPD Assessment Test (CAT), Exacerbations of Chronic Pulmonary Disease Tool (EXACT), and Malnutrition Universal Screening Tool (MUST) will be administered in accordance with their user instructions. Spirometric measurements will be performed in accordance with published guidelines (British Thoracic Society COPD Consortium, 2005).[72] The McKenzie skin blanch test will be as perfomed as described previously.[73,74] Blood glucose measurements will be taken using a CE-marked device licensed for this purpose.

9.8.1 Laboratory procedures

Routine blood samples for analysis in the hospital laboratory will be taken as part of standard clinical care and will be handled accordingly.

Blood samples taken for the purpose of future serum analyses will be taken directly to the study team laboratory (on-site), where samples will be centrifuged and a serum samples obtained. These will be divided into aliquots and stored at -80°C. The resulting acellular samples will be retained for future measurement of inflammatory cytokines and markers of oxidant–antioxidant balance, subject to necessary ethical and institutional approvals.

9.9 Definition of end of trial

This trial will end when the last patient attended for their follow-up appointment, or when the timeframe for this has elapsed and reasonable steps have been taken to contact the participant, to perform the follow-up assessments and to ensure the study treatment has been discontinued or, if necessary, destroyed. A few participants may not yet have received their 12-week

telephone, postal and electronic follow-up at this time. This will be undertaken as per the protocol; however, no new participants will be enrolled, and no study treatment will be prescribed, dispensed or administered, following declaration of the end of the trial.

9.10 Discontinuation/withdrawal of participants and stopping rules

9.10.1 Subject withdrawal procedure

Subject will be withdrawn from the study if they are unable or unwilling to continue the study treatment and/or follow-up arrangements.

For patients who withdraw from the study, consent will be sought to analyse the data and samples already collected. Patients withdrawn from the study will be asked to attend for follow-up as per the protocol. Patients who choose not to attend follow-up will be asked whether they can be contacted by telephone and post to establish their health status and whether any further exacerbations have occurred.

In the event that a subject is withdrawn during the study enrolment period, attempts will be made to enrol a replacement subject.

9.10.2 Treatment suspension and stopping rules

The study treatment will be suspended or stopped under the following criteria.

**Criterion**	**Definition**	**Criteria under which resumption of study treatment may be considered**
Potential adverse reactions to the study treatment:		
Allergic reaction to study treatment	Any clinical reaction considered likely to represented an immune-mediated reaction to the study treatment	None
Minor side effect(s) (see also section 10.4, dose modifications)	Any side effect thought to be attributable to the study treatment, which causes distress or discomfort.	Depending on the severity of the reaction, and the wishes of the patient, this may lead to treatment suspension or dose-reduction. Subsequent resumption of treatment will be considered when the reaction has subsided
Elevated plasma lactate concentration	Plasma lactate concentration ≥3.0 mmol/L, with or without a co-existing abnormality that is considered likely to be the cause of the rising lactate concentration	If there was no alternative likely cause for the elevated lactate concentration, the study treatment will not be restarted. If there was a credible alternative explanation, consideration will be given to the resumption of the study treatment when the precipitant has resolved and the plasma lactate concentration is <3.0 mmol/L
Hypoglycaemia (in association with concomitant glucose-lowering treatment)	Blood glucose <3.3 mmol/L (near-patient capillary measurement or laboratory measurement), judged likely to be an adverse reaction to concomitant glucose-lowering therapy	Euglycaemia for at least 2 hours

Hypoglycaemia (in the absence of concomitant glucose-lowering treatment)	Blood glucose <3.3 mmol/L (near-patient capillary measurement or laboratory measurement), in the absence of, or considered unlikely to have been caused by, concomitant glucose-lowering therapy	None
Significant overall clinical deterioration:		
Admission to critical care unit	Admission of the patient to the any intensive care or high dependency unit or a plan to do so imminently	None
Major irreversible deterioration	A clinical deterioration from which the patient is deemed unlikely to survive to hospital discharge	None
Risk factors for lactic acidosis and/or hypoglycaemia:		
Decompensated hypercapnic respiratory failure	Partial pressure of carbon dioxide in arterial blood higher than the upper limit of normal, with arterial blood pH below lower limit of normal	Resolution of acidosis and no requirement for assisted ventilation for at least 48 hours.
Severe sepsis	As defined in section 7.2 (exclusion criteria)	Resolution of the episode of severe sepsis, and clinical stability for at least 48 hours, as determined by the study investigators, with input from the treating physicians as necessary
Metabolic acidosis	An arterial or venous blood pH below the lower limit of normal and an arterial or venous bicarbonate concentration (measured or calculated) below the lower limit of normal.	Resolution of the metabolic acidosis, and clinical stability for at least 48 hours, as determined by the study investigators, with input from the treating physicians as necessary (provided the criteria for discontinuation due lactate accumulation or acidosis are not met, see below)
Renal impairment	Estimated glomerular filtration rate (eGFR) <45 mL/minute/1.73 m^2 or serum creatinine >130 µmol/L	Estimated GFR ≥45 mL/minute/1.73 m^2 or serum creatinine ≤130 µmol/L, and it is judged that the cause of the deterioration has resolved and is unlikely to recur
Hepatic insufficiency	A clinical suspicion of liver disease in conjunction with a serum bilirubin concentration >34 µmol/L	None
Alcohol withdrawal syndrome	Clinical diagnosis of alcohol withdrawal syndrome	None
Acute coronary syndrome	Diagnosis of acute coronary syndrome by the treating physicians and/or the initiation of treatment with aspirin, clopidogrel and heparin (low molecular weight or unfractionated, at a dose recommended for the treatment of acute coronary syndrome)	The diagnosis is refuted (for example, on the basis of serial cardiac troponin measurements, or following review by a cardiology specialist)
Severe cardiac failure	Clinical diagnosis of cardiac failure by the treating physicians and/or investigator on the basis of typical symptoms and/or signs and/or results of investigations, which is leading to shortness of breath at rest	None

Radiological examination requiring the administration of intravenous contrast material	Any radiological examination requiring the administration of intravenous contrast material	No earlier than 48 hours after contrast administration, if the eGFR or serum creatinine concentration is within 15% of the pre-examination value, and greater than >45 mL/minute/1.73 m^2 or ≤130 µmol/L, respectively
Oral nutritional intake suspended	A 'nil by mouth' order or equivalent	Discontinuation of this order and resumption of oral intake
Substantially-reduced caloric intake	Judged by the investigator and/or clinical care team (including a dietetic specialist, where requested), on the basis of clinical history, collateral history, clinical observation, and food charts	Resumption of adequate caloric intake, as judged by the investigator, with advice from the clinical care team
Hospital readmission	A participant who, following discharge, is re-admitted to any hospital, and either (a) another stopping or suspension criterion is met, or may have been met; or (b) the investigator has concerns that the protocol cannot be safely continued (for example, if the participant is admitted to a hospital elsewhere)	When the investigator has reviewed the participant in person to ensure that no stopping/suspension criteria have been met, and that their continued participation in the trial will be safe.
Requirement for open-label glucose lowering therapy (other than insulin)	Treating clinician judges that open-label treatment with a glucose-lowering agent would be important to the participant's ongoing clinical management (unless permitted under the study protocol—that is, open-label insulin administration during the inpatient phase)	None
Withdrawal of consent:		
Participant withdraws consent	Participant reports to a member of the clinical or study team that they wish to permanently discontinue the study treatment and/or their involvement in the clinical trial	None

If a discontinuation occurs during the enrolment period, attempts will be made to recruit a substitute patient.

10 Name and description of all drugs used in the trial

10.1 Name, description and source of each IMP and placebo

Metformin 500 mg capsules will be sourced from TEVA UK Ltd and supplied to Bilcare GCS (Europe) LTD (MIA IMP license number 10284).

Bilcare will over-encapsulate metformin 500 mg capsules and provide matched placebo capsules. The resulting metformin 500 mg capsule will appear identical in weight, colour and size to the placebo capsules

Metformin/placebo 500 mg capsules will be packaged into containers to hold sufficient doses for the patient to complete 35 days of prescribed treatment according to the research protocol.

The product labels will be fully compliant with both Annexe 13 of the Rules and Guidance for Pharmaceutical Manufacturers and Distributors (Orange Guide) and the protocol.

Metformin capsules and matched placebo will be stored at ambient room temperature until the expiration/retest date allocated by Bilcare.

10.2 Accountability procedures for the investigation product, including the placebo

The study treatment will be received by a designated person at St George's Hospital Pharmacy, and handled and stored safely and securely in a location only accessible by authorised personnel. Upon receipt, all study IMP will be stored according to instructions specified on the drug labels. Clinical supplies are to be dispensed only in accordance with the protocol. Supplies for patients recruited at the other site(s) will be transferred at ambient temperature and with all required records of dispatch, transfer conditions, and receipt.

The pharmacy will be responsible for maintaining an accurate record of the shipment and dispensing of study IMP in the accountability logs. Patients will be requested to return all unused IMP(s) and packaging at the end of their prescribed study period to the Clinical Trials team. The Clinical Trials team will be responsible for maintaining & updating the drug accountability log with returned IMP, in the hospital pharmacy file.

At the conclusion of the study (or at regular agreed intervals for patient returned IMPs) all study supply accountability records will be cross-checked with remaining study IMP to ensure full accountability before drug destruction.

Drug destruction will be conducted, once agreed by the sponsor and in accordance to local hospital policy, and this will be documented on the drug destruction log in the pharmacy study file.

10.3 Route of administration, dosage, dosage regimen, and treatment period(s) of the IMPs

The study medicine will be taken orally for 28-35 days. The target dosage will be two capsules taken twice daily with meals (metformin 2000 mg/day or placebo). To minimise the risk of gastrointestinal upset, participants will be advised to take the study treatment soon after meals (typically within 20 minutes), and the dose will be escalated as follows:

- Day 1: 500 mg taken after food following first mealtime after randomisation
- Day 2: 500 mg taken after breakfast time and 500 mg after evening mealtime
- Day 3: 1000 mg taken after breakfast time and 500 mg after evening mealtime
- Day 4 until follow-up (day 28–35): 1000 mg taken after breakfast time and 1000 mg after evening mealtime

10.4 Dosage modifications

In the event that a patient suffers mild gastrointestinal side effects (including, but not limited to, diarrhoea, nausea, vomiting, abdominal discomfort, and taste disturbance), headache, or weakness, the drug will be continued provided the side effects are mild and tolerable. If the side effects are not tolerable, treatment with the study drug will either be reduced in dosage (to the last tolerated dosage), or suspended, depending on the severity of the side effect, the degree of discomfort it has caused, and the wishes of the patient.

Following dose-reduction or suspension of the study treatment, one attempt will be made to re-introduce the drug and/or increase it to target dose, after resolution of the side effects. The timing of this will be judged on a case-by-case basis. If on this attempt the drug cannot be tolerated at any dose due to recurrence of side effects, it will be discontinued. If the drug can be tolerated at a sub-maximal dosage, then it will be continued at this lower dosage until study completion.

All decisions on dose modification will be made or confirmed by a medically-qualified member of the study team.

10.5 Assessment of compliance

During the inpatient phase, administration of the study treatment will be supervised and documented by nursing staff. In the event that at the study treatment is not administered, the reason will be recorded in the study folder.

During the outpatient phase, compliance will be assessed by pill counts and by structured interview.

10.6 Post-trial IMP arrangements

The study treatment will be stopped on completion of the treatment period. No provisions have been made to continue the treatment beyond this period, as it is unlikely that this would be clinically indicated. However, if information collected in the study suggested that the patient may have undiagnosed and/or undertreated diabetes mellitus, the patient, and with their

consent, their general practitioner, will be informed of this such that further assessment and treatment may be instituted.

10.7 Name and description of each NIMP

All patients regardless of allocation to metformin or placebo treatment will be prescribed prednisolone 30 mg daily, administered orally, for a minimum of 7 days. This is consistent with current guidelines for the management of patients requiring hospitalisation for COPD exacerbations.[4] Prednisolone will be routinely prescribed and obtained from normal hospital stocks. There are no special labelling requirements over and above the normal Pharmacy labelling SOP. Any suspected Adverse Drug Reactions or side effects that may be attributable to the administration of prednisolone will be reported through the Yellow Card system.

The McKenzie skin blanch test will be performed using beclometasone diluted in ethanol, administered by topical application. Confirmation that beclometasone used in this context is considered a NIMP has been obtained from the Medicines and Healthcare Products Regulatory Agency Clinical Trials Helpline.

11 Pharmacovigilance

11.1 Definitions

Adverse Event (AE)—any untoward medical occurrence in a patient or clinical trial subject administered an IMP and which does not necessarily have a causal relationship with this treatment (i.e. any unfavourable or unintended change in the structure (signs), function (symptoms), or chemistry (lab data) in a subject to whom an IMP has been administered, including occurrences unrelated to that product).

Adverse Reaction (AR)—any untoward and unintended responses to an IMP related to any dose administered (i.e. any unfavourable or unintended change in the structure (signs), function (symptoms), or chemistry (lab data) in a subject to whom an IMP has been administered and related to any dose administered).

Serious Adverse Event (SAE) or Serious Adverse Reaction (SAR)—any Adverse Event or Reaction in a trial subject that:

- Results in death; or
- Is life-threatening (places the subject in the view of the investigator at immediate risk of death)
- Requires hospitalisation or prolongation of existing hospitalisation (hospitalisation is defined as an inpatient admission, regardless of length of stay; even if it is a precautionary measure for observation; including hospitalisation for an elective procedure, for a pre-existing condition)
- Results in persistent or significant disability or incapacity (substantial disruption of one's ability to conduct normal life functions)

- Consists of a congenital anomaly or birth defect (in offspring of subjects or their parents taking the IMP regardless of time of diagnosis).

Important medical events that may not be immediately life-threatening or result in death or hospitalisation but may jeopardise the subject or may require intervention to prevent one of the outcomes listed in the definition of serious will also be considered serious.

Suspected Unexpected Serious Adverse Reaction (SUSAR)—an Adverse Reaction which is classed in nature as both serious and unexpected.

Unexpected adverse reaction means an adverse reaction the nature and severity of which is not consistent with the information about the medicinal product in question set out in the Summary of Product Characteristics,[36] the latest edition of the British National Formulary, and/or in this protocol.

11.2 Recording Adverse Events (AEs)

All Adverse Events will be recorded in the hospital notes in the first instance.

If the Investigator suspects that the disease has progressed faster due to the administration of the IMP, then he will report this as an unexpected adverse event to the Sponsor.

Clinically significant abnormalities in the results of objective tests (*e.g.* laboratory variables) may also be recorded as Adverse Events.

Metformin has been in common use for over 50 years. Considerable experience has been amassed regarding its risk profile in diabetes mellitus, polycystic ovary syndrome, and in a wide range of co-incidental diseases. These data have been gathered both from clinical practice and more than 70,000 patient-years of exposure within clinical trials.[41] Although this study concerns the use of metformin outside of its licensed indication, and which has not previously been studied in the clinical trial setting, it is not novel in clinical practice. An audit conducted in this hospital indicated that approximately one-third of patients hospitalised with COPD and diabetes mellitus are treated with metformin, and it is continued after admission in approximately three-quarters of these. We feel that this practice is likely to be representative of practice elsewhere. As such, our assessment is that the safety profile of metformin in this context is well-characterised. Intensive recording and notification of all Adverse Events is extremely unlikely to reveal any previously unrecognised adverse reactions.

11.3 Procedures for recording and reporting SAEs

The following descriptions will be used to record AEs:

Clinical symptoms—a simple and brief description.

Severity will be described using following categories:

- **Mild**—the adverse event does not interfere with the volunteer's daily routine, and does not require intervention; it causes slight discomfort.

- **Moderate**—the adverse event interferes with some aspects of the volunteer's routine, or requires intervention, but is not damaging to health; it causes moderate discomfort.
- **Severe**—the adverse event results in alteration, discomfort or disability which is clearly damaging to health.

Relationship to treatment—the assessment of relationship of AEs to the administration of IMP is a clinical decision based on all available information at the time of the completion of the CRF. The following categories will be used:

- **Definitely**—there is clear evidence to suggest a causal relationship, and other possible contributing factors can be ruled out.
- **Probably**—there is evidence to suggest a causal relationship, and the influence of other factors is unlikely.
- **Possibly**—there is some evidence to suggest a causal relationship (e.g. the event occurred within a reasonable time after administration of the trial medication). However, the influence of other factors may have contributed to the event (i.e. the patient's clinical condition, other concomitant events).
- **Unlikely**—there is little evidence to suggest there is a causal relationship (e.g. the event did not occur within a reasonable time after administration of the trial medication). There is another reasonable explanation for the event (e.g. the patient's clinical condition, other concomitant treatments).
- **Not related**—there is no evidence of any causal relationship.
- Not Assessable

Expectedness, where applicable, will be assessed for Adverse Reactions according to the following definitions:

- **Expected**—an Adverse Reaction that is consistent with the information about the IMP listed in the SmPC.
- **Unexpected**—an Adverse Reaction that is not consistent with the information about the IMP listed in the SmPC.

Collection, recording and reporting of AEs (including serious and non-serious events and reactions) to the Sponsor will be done according to Sponsor's Pharmacovigilance SOP.

All SAEs will be recorded in the hospital notes and the CRF, and the sponsor's SAE Recording Log. The SAE Log will be sent to Sponsor on request and every 2 months.

All SAEs will be reported to the Sponsor via the JRO on an SAE form unless stated in the protocol that some expected SAEs will not be reported to the Sponsor, with justification as to why they will not be reported.

The Chief or Principal Investigator will complete the Sponsor's SAE form and the form will be faxed to the JRO on 020 8725 0794 or e-mailed to adverseevents@sgul.ac.uk, within 24hrs of his/her becoming aware of the event.

The Chief or Principal Investigator will respond to any SAE queries raised by the Sponsor as soon as possible.

All SUSARs will be notified to the Sponsor immediately or at least within 24hrs of the Investigator becoming aware of the event.

11.4 Protocol Defined Events (PDEs)

This trial involves patients hospitalised for exacerbations of COPD, a condition that is associated with high morbidity and mortality. The in-hospital mortality rate has been reported to range between 4% and 30%. In an audit of a subset of patients with COPD (those who also had diabetes mellitus), 14/108 (13%) required non-invasive ventilation for respiratory failure, and 17/108 (16%) died in-hospital or within 30 days of discharge.

Many important and potentially Serious Adverse Events (SAEs) are common in the population under study, which are unlikely to be related to the IMP, but rather the underlying disease or co-morbidities and these will be identified and reported as Protocol Defined Events. A list of anticipated PDEs is presented below. The occurrence of any of the events listed below will be documented in the patients hospital notes, CRFs and Sponsor's AE Log. The PDEs will not be reported to Sponsor immediately using the SAE Reporting Form. However, the Sponsor will be made aware on regular review of the completed AE Log through submission to the JRO on request or every 2 months. Similarly, an assessment of expectedness will not be required unless they fall within the definition of an Adverse Reaction.

- Failure to recover to baseline
- Recurrent exacerbation of COPD
- Pneumonia
- Respiratory failure
- Sepsis

For the avoidance of doubt, a Protocol Defined Event remains as such even if it has an attribute that defines it at a Serious Adverse Event (for example, a recurrent exacerbation of COPD requiring hospital admission should be treated as a Protocol Defined Event).

11.5 Notification of deaths

All deaths, including deaths deemed unrelated to the IMP, even if they occur earlier than expected will be reported to the Sponsor.

This report will be sent to the JRO as documented in Section 11.3 within 24 hours of the Chief or Principal Investigator (CI/PI) becoming aware of the death.

11.6 Reporting of SUSARs

The JRO will notify all SUSARs to the MHRA and the main REC using CIOMs form. The CIOMs form should be completed by the CI and PI at each site and sent to the JRO along with the Sponsor's SAE Recording and Reporting Form as described in Section 11.3 of the protocol. The

JRO will inform the MHRA and the main REC of fatal or life threatening SUSARs as soon as possible, but no later than 7 calendar days after the JRO receives and dates the SAE report form. Any additional information will be reported within 8 days of sending the first report.

The JRO must report all other SUSARs and safety issues to the MHRA and main REC, as soon as possible but no later than 15 calendar days after the JRO has first knowledge of the minimum criteria for expediting reporting.

11.7 The type and duration of the follow-up of subjects after AEs

Follow-up will continue for the duration of the study.

Any participant suffering an AE while an inpatient, will receive assessment, treatment and follow-up by the nursing and clinical care team, and as required by the study team, until clinical stability is resumed and/or the AE has resolved. Ongoing care and follow-up will be provided by the nursing and clinical team, with assistance of the study team as required.

A 24-hour contact number will be available for all patients and/or clinical staff to contact the study team if required.

Patients will be given advice to contact the study team (using a 24-hour contact number), or if need be, the ambulance service, should they suffer an AE following discharge. Depending on the nature of the reaction, advice may be given by telephone that no action is required; that the participant should seek urgent medical review (in which case the study team endeavour to make contact with the relevant receiving unit); that the participant should attend for early follow-up with the study team (and as appropriate, that the study treatment should be suspended). Follow-up will be provided by the study team and/or the participant's general practitioner (in liaison with the study team), with an intensity appropriate to the nature and severity of the adverse reaction.

The JRO will ensure that all SAE/Rs reported to Sponsor will be followed every 2 weeks until resolution, and in accordance with Sponsor's PV SOP.

11.8 Development Safety Update Reports (DSURs)

The CI will prepare the DSUR, according to the ICH Guideline E2F – "Note for guidance on development safety update reports". It will be reviewed by the Sponsor and when necessary be referred to an independent committee (i.e. Research Governance Safety Committee). The JRO will provide the main REC and the MHRA with the DSUR within 60 days of the anniversary of the CTA authorisation.

11.9 Annual Progress Reports (APRs)

The Chief Investigator will prepare the APR. It will be reviewed by the JRO and sent to the main REC within 30 days of the anniversary date on which the favourable opinion was given, and annually until the trial is declared ended.

11.10 Pregnancy

Metformin is not known to be harmful in pregnancy; animal studies do not indicate harmful effects with respect to pregnancy, embryonic or foetal development, parturition or postnatal development.[36] Although not licensed for this indication, the British National Formulary lists metformin as a treatment option for both pre-existing and gestational diabetes in pregnancy.[39] Consequently, it seems extremely unlikely that metformin presents significant hazards in relation to the unborn child. Moreover, the epidemiology of chronic obstructive pulmonary disease is such that participants are very unlikely to be of childbearing age; most patients with COPD are over 65-years-old.[4]

Nevertheless, the pregnancy risk of female patients of potentially childbearing age will be ascertained by establishing their menstrual history; past symptoms suggestive of menopause; and, as appropriate, contraceptive use. Women who are of childbearing age and who are fertile will be asked to use medically-effective contraceptive methods while they are enrolled in the study. They will be asked to stop taking the study medicine if their period is more than one week overdue of if they otherwise suspect they are pregnant, and to report this to the study team. The study treatment will be discontinued and details of the patient's involvement in the study will be passed to the relevant obstetric team, if the patient was assigned to active treatment. In view of animal data suggesting the absence of teratogenicity, in conjunction with the absence of clinical data suggesting teratogenicity in the face of considerable experience in its use (including in polycystic ovary syndrome as a means to improve fertility, and in diabetes in pregnancy), we do not propose to offer further follow-up to the offspring of a participant in this trial.

All pregnancies will be reported to the JRO using the Pregnancy Reporting Form and followed up in accordance with the JRO Pharmacovigilance SOP.

11.11 Reporting urgent safety measures

Regulation 30 of the Medicines for Human Use (Clinical Trials) Regulations 2004 [Statutory Instrument 2004/1031], as amended by Statutory Instrument 2006/1928 states "the Sponsor and the Investigator may take appropriate urgent safety measures in order to protect the subjects of a clinical trial against any immediate hazard to their health or safety. If measures are taken, the Sponsor shall immediately and in any event no later than 3 days from the date the measures are taken, give written notice to the MHRA and the relevant REC of the measures taken and the circumstances giving rise to those measures."

In order to prevent any delays in the reporting timelines the Sponsor has delegated this responsibility to the CI/PI. Therefore the CI/PI must report any urgent safety measures to the MHRA directly, and in parallel to the Sponsor.

Please refer to the following website for details on clinical trials safety reporting: http://www.mhra.gov.uk/Howweregulate/Medicines/Licensingofmedicines/Clinicaltrials/Safetyreporting-SUSARSandASRs/index.htm

11.12 Notification of serious breaches to GCP and/or the protocol

Any Protocol Deviations, Violations, Potential Serious Breaches and urgent safety measures will be recorded using Sponsor's Log issued during Sponsor's Trial/Site Initiation meeting/visit.

Regulation 29A of the Medicines for Human Use (Clinical Trials) Regulations2004 [Statutory Instrument 2004/1031], as amended by Statutory Instrument2006/1928, contains a requirement for the notification of "serious breaches" of GCP or the trial protocol:

(1) The Sponsor of a clinical trial shall notify the licensing authority in writing of any serious breach of

(a) the conditions and principles of GCP in connection with that trial; or

(b) the protocol relating to that trial, as amended from time to time in accordance with regulations 22 to 25, within 7 days of becoming aware of that breach.

(2) For the purposes of this regulation, a "serious breach" is a breach which is likely to effect to a significant degree

(a) the safety or physical or mental integrity of the subjects of the trial; or

(b) the scientific value of the trial.

The Sponsor will be notified immediately of any case where the above definition applies during the trial conduct phase. The sponsor's SOP on the Protocol Violation/Deviations and Serious Breaches will be followed.

12 Data management and quality assurance

12.1 Confidentiality

All data will be handled in accordance with the Data Protection Act 1998.

The Case Report Forms (CRFs) will not bear the subject's name or other personal identifiable data. The subject's initials, date of birth and trial identification number will be used for identification.

All data pertaining to identifiable persons shall be regarded as confidential. While a patient is enrolled in the study, and remains a hospital inpatient, data in relation to their nutritional assessment, administration of the study treatment (blinded), capillary glucose measurements and safety parameters, shall be available to the clinical team caring for the patient. This is because such data may be important to the patients' clinical care, and this may avoid unnecessary duplication.

12.2 Data collection tool

Case report forms (CRFs) will be designed and produced by the investigator. The final version will be approved by the Sponsor. All data will be entered legibly in black ink with a ball-point pen. If an error is made, the error will be crossed through with a single line in such a way that the original entry can still be read. The correct entry will then be clearly inserted, and the alterations will be initialled and dated by the person making the alteration. Overwriting or use of correction fluid will not be permitted.

It will be the responsibility of the investigator to ensure the accuracy of all data entered in the CRFs. The delegation log will identify all those personnel with responsibilities for data collection and handling, including those who have access to the trial database.

The Case Report Forms will be designed to capture the data required to inform baseline comparisons of the groups, record safety parameters, and provide data for analysis of primary, secondary and safety endpoints as described in the protocol and statistical plan. Standard abbreviations will be used for missing data (NA, not available/applicable; ND, not done; UNK, unknown).

12.3 Data handling and analysis

Data relevant to patients' clinical care will be recorded and stored in a study folder, held on the ward, under the guardianship of the ward nursing staff caring for the patient. Data not relevant to the patients' clinical management will be held in a study folder, kept in a secure location away from the ward. All person-identifiable data held away from the ward—both during and after the study period—will be kept in a locked room within a card-accessed area of the University site, which is protected by 24-hour on-site security personnel and fire detection and alarm system.

Data will be transferred to an electronic database in the Excel software programme by double entry. The resulting databases will be compared to identify discrepancies, and subject to range checks to identify missing and spurious data. Such data will be corrected with reference to the Case Report Forms and, as necessary, to source documents (original clinical records, electronic records, observation records, electronic device records for capillary glucose and exacerbation of chronic pulmonary disease tool (EXACT) measurements, photographic records of skin blanch test results).

Electronic data will be kept in encrypted databases, on a restricted access area of the University computer network. This is backed up locally on a daily basis, and monthly backups are held securely in an offsite location. No patient identifiable data will be stored on laptop computers or portable storage devices, or will be sent by electronic mail. The computer network is protected by an intrusion detection system.

13 Archiving arrangements

The trial documents (including trial master file, case report forms and consent forms) will be kept for a minimum of five years. They will be stored in locked offices within the St George's, University of London site, or in an archiving site as recommended and/or approved by the Joint Research Office. The trial database will be kept electronically on the St George's, University of London computer network, for a minimum of five years.

14 Statistical methods

14.1 Statistical input in trial design

The trial design and statistical analysis plan have been constructed with input from Dr Jan Poloniecki, Senior Lecturer in Medical Statistics, St George's, University of London.

14.2 Endpoints

14.2.1 Primary endpoint

Mean capillary glucose concentration during hospitalisation period—defined as the mean of all capillary glucose measurements obtained according to the study protocol for that patient during the period between enrolment in the trial and hospital discharge. This measure has been evaluated against more complex endpoints (time-averaged glucose and hyperglycaemic index) and found to be the most practical metric of hyperglycaemia-associated risk.[13]

14.2.2 Secondary endpoints

Unless otherwise specified, the endpoints described below related to a comparison of data between the active treatment and placebo groups.

Clinical efficacy:

- COPD Assessment Test score at 4 weeks and, where available, at 12 weeks—defined as the sum of the points marked on the patient-completed COPD Assessment Test, as published; comparisons of mean score at follow-up and proportion of patients with improvement ≥5 points
- Exacerbation of Chronic Pulmonary Disease Tool (EXACT) scores—mean scores at days 5, 10 and 28
- time to medical fitness for discharge—defined as the number of days between study enrolment and the day on which the patient is documented by the clinical care team to be medically fit for hospital discharge, if this is different to the date of actual discharge
- time to actual discharge—defined as the number of days between study enrolment and the day on which the patient is discharged from the hospital
- rates of recurrent exacerbation, readmission, or death—defined, respectively as the number of days between study enrolment and the day on which the patient is prescribed a systemic antibiotic or steroid (for an exacerbation of COPD), is readmitted to hospital (for any reason except planned elective treatment or investigation), or dies (from any cause)

Glycaemic control:

- mean daily insulin use during hospitalisation period—defined as total number of units of insulin administered (of any formulation), divided by the duration of hospitalisation (in days)
- mean HbA1c and serum fructosamine concentration at follow-up, and change from baseline

Inflammation and infection:

- mean C-reactive protein concentration at discharge and follow-up (absolute values and change from baseline)
- a relevant panel of markers of inflammation and oxidative stress, measured on serum samples collected at study entry, discharge and follow-up (absolute values and change from baseline)

Other metabolic effects:

- mean body mass index at follow-up
- mean waist circumference at follow-up

Airflow limitation

- FEV_1 at 4 weeks—mean values at follow-up and mean change from discharge

14.2.3 Feasibility, safety and tolerability assessment

Feasibility of the treatment strategy will be assessed with reference to the proportion of scheduled doses that were administered. Safety will be assessed with reference to plasma

lactate concentration and records of adverse events. Tolerability will be assessed with reference to records of adverse events.

14.3 Sample size and recruitment

14.3.1 Sample size calculation

In an audit and service evaluation study conducted in this centre (unpublished data, available from PI), the case notes of 20 patients hospitalised for COPD exacerbations were evaluated retrospectively. A median of 2 capillary glucose measurements were made per patient (interquartile range 1.8–3.3). The measurements had a mean value of 7.7 mmol/L and standard deviation 1.5 mmol/L. Based upon these data, a sample size of 44 (randomised equally to treatment and control groups) would allow the detection of a difference in the mean glucose concentrations between the two groups of 1.5 mmol/L with 90% power (using the *t*-distribution with a two-sided alpha of 0.05). This difference would be clinically significant and, on the basis of prior observational data,[14] might correspond to a difference in the risk of adverse outcome (death or prolonged hospitalisation) of 22.5%. In the initial calculation of sample size, provision was made for an expected drop-out rate of approximately 5%, resulting in a target recruitment size of 46 patients. With effect from Substantial Amendment 4 approval, the target sample size is increased to 69 patients, with a change in the active:placebo allocation ratio from 1:1 to 2:1 (maintaining stability of the control group size). This is to permit a fuller assessment of the feasibility, safety and tolerability of the drug when used in COPD exacerbations in accordance with the accelerated dose-escalation regimen. Under conditions of 2:1 allocation, the minimum number of participants required to maintain statistical power for the primary endpoint analysis (as defined above) is 48.

14.3.2 Planned recruitment rate

There are approximately 400 admissions per year to St George's Hospital of patients with COPD exacerbations. Assuming that approximately 25% are potentially eligible and willing to participate, it is anticipated that patients will be recruited at a rate of approximately 2 per week. This should allow the target sample to be recruited in 23 weeks. It should be noted that there is seasonal variation in the rate of admissions for COPD, with a peak in the winter months. The study timetable allows for an enrolment period of up to 9 months, and it is planned that recruitment will commence before winter.

14.4 Statistical analysis plan

14.4.1 Summary of baseline data and flow of patients

The report will detail the number of eligible patients for the trial, the number consenting and the number randomised. A breakdown will be presented for each group of the numbers of participants assigned, receiving the intended treatment, completing the study protocol, and analysed for the primary outcome. This information will be displayed as a flow diagram.

The following data will be used to describe baseline comparability of study groups:

- Age (continuous)—mean and standard deviation

- Sex (categorical)—proportions
- COPD Assessment Test score (interval scale)—mean and standard deviation
- Exacerbation of Chronic Pulmonary Disease Tool score (interval scale)—mean and standard deviation
- Forced expiratory volume in 1 second (continuous)—mean and standard deviation
- Presence of radiographic consolidation (categorical, defined by the presence or absence of consolidation or airspace shadowing on a chest X-ray, as reported by a radiology specialist as part of routine clinical practice)—proportions
- Venous blood glucose concentration (continuous)—mean and standard deviation
- Haemoglobin A1c (continuous)—mean and standard deviation
- C-reactive protein concentration (continuous)—mean and standard deviation

14.4.2 Primary endpoint analysis

The primary analysis will be performed in accordance with the intention to treat principle. For comparison, a per protocol analysis will be provided as a secondary outcome measure. Statistical significance shall be defined as a two-sided p-value <0.05. Any post hoc analyses will be defined as such in the research report. No interim analyses are planned.

The primary endpoint for this study will be the mean hospitalisation glucose concentration. The mean of the individual mean values recorded among participants allocated to active treatment will be compared with the mean of the individual mean values recorded among patients allocated to placebo treatment. The significance of the observed difference will be tested using an independent sample t-test.

14.4.3 Secondary endpoint analysis

This study has not been powered for its secondary endpoints. The secondary analyses are for the purpose of hypothesis generation, rather than to provide firm conclusions. This study will inform the design of a future larger trial.

Means will be compared using the t-test (for normally-distributed continuous data), Mann-Whitney test (for non-normally-distributed continuous data), or Chi-square or Fisher's exact test as appropriate (for categorical data). Time-to-event data will be analysed and presented using Kaplan-Meier curves, the log rank test, and Cox proportional hazards regression.

14.4.4 Sub-group analyses

A single subgroup analysis is planned, based on the presence or absence of radiographic consolidation on the admission chest radiograph. The presence of radiographic consolidation shall be defined as a report by a radiology specialist of definite or likely consolidation or airspace shadowing on the first chest radiograph obtained during the hospitalisation period, which was not present on previous chest radiographs (if available), and for which pneumonia is a differential diagnosis. Where no chest radiograph has been performed, it shall be assumed that consolidation is absent (it is considered extremely unlikely that a patient admitted for COPD would not have a chest radiograph performed at or soon after admission).

The presence of radiographic consolidation is thought to identify patients with a different clinical phenotype and prognosis. As such, the primary and secondary analyses will be repeated in those patients who do, and do not, have evidence of radiographic consolidation. These will be considered as secondary endpoints and regarded as hypothesis-generating.

14.4.5 Sensitivity and other planned analyses

None planned.

14.5 Randomisation

Prior to Substantial Amendment 4 approval, participants were allocated to metformin 500 mg capsules or matched placebo by equal group random allocation in blocks of four at Chelsea and Westminster Hospital and blocks of six at St George's Hospital and other sites. Since Substantial Amendment 4 approval, allocation to metformin or placebo will be in a 2:1 ratio in blocks of six. In light of the fact that recruitment commenced under conditions of 1:1 active:placebo allocation, and shall complete under conditions of 2:1 allocation, it is understood that the final allocation ratio cannot be predicted exactly. However, a review of the allocation ratio for participants recruited prior to Substantial Amendment 4 (1.75:1) indicates that the final ratio will be approximately equal to, and no greater than, 2:1. As such, it is judged that there is no need to apply a correction for prior participant allocation (the assessment of prior participant allocation was performed by the IMP manufacturer, at the request of the investigators, without revealing any data to investigators that might unblind them to individual or site-specific allocation data).

The randomisation list is produced electronically by the drug manufacturer (Bilcare [GCS] Europe Ltd). A fresh supply of IMP and placebo will be used from approval of Substantial Amendment 4, which will have been randomised according to the revised specifications detailed above.

14.6 Interim analysis

There are no planned interim analyses. No criteria have been set for early termination of the trial on the basis of efficacy/effectiveness outcome measures. The trial will be terminated early on safety grounds if any case of lactic acidosis occurs in the active treatment group (defined as a blood lactate concentration ≥5 mmol/L and an arterial blood pH below the lower limit of normal), which is judged to be definitely or probably related to the study treatment.

14.7 Other statistical considerations

The statistical analysis plan shall be finalised prior to enrolment of the first patient. Any substantive changes or deviations made after this time, will be reported and justified in the protocol and/or the final report as appropriate.

15 Committees in involved in the trial

This trial will have a Trial Steering Committee (TSC). The membership and terms of reference for this committee will be provided separately.

16 Direct access to source data

The investigator(s)/institution(s) will permit trial-related monitoring, audits, REC review, and regulatory inspection(s), providing direct access to source data/documents. Trial participants are informed of this during the informed consent discussion. Participants will consent to provide access to their medical notes.

17 Ethics and regulatory requirements

The sponsor will ensure that the trial protocol, Patient Information Leaflet (PIL), Informed Consent Form (ICF), GP letter and submitted supporting documents have been approved by the MHRA and a main Research Ethics Committee (REC), prior to any patient recruitment taking place. The protocol and all agreed substantial protocol amendments, will be documented and submitted for ethical and regulatory approval prior to implementation.

Before sites can enrol patients into the trial, the Principal Investigator must apply for Site Specific Assessment from Trust Research & Development (R&D) and be granted written NHS R&D approval. It is the responsibility of the Principal Investigator at each site to ensure that all subsequent amendments gain the necessary approval. This does not affect the individual clinician's responsibility to take immediate action if thought necessary to protect the health and interest of individual patients (see section 11.11 for details of reporting procedures).

Within 90 days after the end of the trial, the CI and Sponsor will ensure that the main REC and the MHRA are notified that the trial has finished. If the trial is terminated prematurely, those reports will be made within 15 days after the end of the trial.

The CI will supply a summary report of the clinical trial to the MHRA and main REC within 1 year after the end of the trial.

18 Monitoring plan for the trial

The trial will be monitored in a risk-proportionate approach according to the monitoring plan agreed and written by the Sponsor, based on the internal risk assessment. It is the responsibility of the JRO to determine the monitoring risk assessment and explain the rationale.

The PI or delegate at each site will be required to complete the JRO self-monitoring template when required to the JRO. It is the CI/PI's responsibility to ensure that any findings identified in the JRO monitoring report are actioned in a timely manner and any violations of GCP or the protocol reported to the JRO immediately.

The CI will be provided with a copy of the study monitoring report during each site monitoring visit.

19 Finance

Funds are available internally to finance this trial. The principal investigator is funded to undertake research as part of his NHS post.

20 Insurance and indemnity

St George's, University of London holds insurance to cover participants for injury caused by their participation in the clinical trial. Participants may be able to claim compensation if they can prove that St George's has been negligent. This clinical trial is conducted in hospital and the hospital continues to have a duty of care to the participant of the clinical trial. St George's, University of London will not accept liability for any breach in the hospital's duty of care, or any negligence on the part of hospital employees regardless of the hospital being NHS Trust or not. This does not affect the participant's right to seek compensation via the non-negligence route.

Participants may also be able to claim compensation for injury caused by participation in this clinical trial without the need to prove negligence on the part of St George's, University of London or another party. Participants who sustain injury and wish to make a claim for compensation should do so in writing in the first instance to the CI, who will pass the claim to the Sponsor's Insurers, via the Sponsor's office.

Hospitals selected to participate in this clinical trial shall provide clinical negligence insurance cover for harm caused by their employees and a copy of the relevant insurance policy or summary shall be provided to St George's, University of London, upon request.

21 Publication policy

Data ownership rights will lie with the institution.

The study team is committed to reporting and disseminating results of the study both by internal report and by publication in peer reviewed, scientific journals and/or conference presentations. Copies of published results will be made available to participants should they so wish. In addition, the results of the research will be discussed with the Patient Advisory Group attached to the study, who will advise on how best to make study results available to patients. One potential route is through the 'Breathe Easy' network of the British Lung Foundation.

22 Statement of compliance

The trial will be conducted in compliance with the protocol, GCP and the applicable regulatory requirement(s).

The study conduct shall comply with all relevant laws of the EU if directly applicable or of direct effect and all relevant laws and statutes of the UK country in which the study site is located including but not limited to, the Human Rights Act 1998, the Data Protection Act 1998, the

Medicines Act 1968, the Medicines for Human Use (Clinical Trial) Regulations 2004, and with all relevant guidance relating to medicines and clinical studies from time to time in force including, but not limited to, the ICH GCP**,** the World Medical Association Declaration of Helsinki entitled 'Ethical Principles for Medical Research Involving Human Subjects' (2008 Version), the NHS Research Governance Framework for Health and Social Care (Version 2, April 2005).

This study will be conducted in compliance with the protocol approved by the REC and according to GCP standards and UK Clinical Trials Regulation. No deviation from the protocol will be implemented without the prior review and approval of the Sponsor and REC except where it may be necessary to eliminate an immediate hazard to a research subject. In such case, the deviation will be reported to the Sponsor and REC as soon as possible.

23 References

1 World Health Organisation. The top ten causes of death. Fact sheet No 310 2008. Available: http://www.who.int/mediacentre/factsheets/fs310/en/index.html (accessed 08/03/2010).

2 Department of Health. *On the state of the public health: Annual Report of the Chief* 22 *Medical Officer 2004.* 2005. Available: http://www.dh.gov.uk/en/Publicationsandstatistics/Publications/AnnualReports/DH_4115776 (accessed 22/03/2010).

3 Healthcare Commission. Clearing the air: A national study of chronic obstructive pulmonary disease. Healthcare Commission, 2006. Available: http://www.cqc.org.uk/_db/_documents/COPD_report1_200607272728.pdf (accessed 22/03/2010).

4 National Clinical Guideline Centre. CG101 Chronic obstructive pulmonary disease (update): full guideline. Available: http://guidance.nice.org.uk/CG101/Guidance/pdf/English (accessed 14/07/2010)

5 Global Initiative for Chronic Obstructive Lung Disease. Global strategy for the diagnosis, management and prevention of chronic obstructive lung disease (updated 2009). Available: http://www.goldcopd.org/Guidelineitem.asp?l1=2&l2=1&intId=2003 (accessed 22/03/2010)

6 Seemungal TAR, Donaldson GC, Paul EA, Bestall JC, Jeffries DJ, Wedzicha JA. Effect of exacerbation on quality of life in patients with chronic obstructive pulmonary disease. Am J Respir Crit Care Med 1998;157(5 Pt 1):1418–1422.

7 British Thoracic Society. Burden of Lung Disease Report, 2nd edn 2006. Available: http://www.brit-thoracic.org.uk/library-guidelines/bts-publications/burden-of-lung-disease-reports.aspx#Burden1 (accessed 11/03/10)

8 Wedzicha JA, Seemungal TAR. COPD exacerbations: defining their cause and prevention. Lancet 2007;370:786–96.

9 Niewoehner DE. The Impact of Severe Exacerbations on Quality of Life and the Clinical Course of Chronic Obstructive Pulmonary Disease. The American Journal of Medicine 2006;119(Suppl 10A):S38–S45.

10 Soler-Cataluña JJ, Martínez-García MÁ, Román Sánchez P, et al. Severe acute exacerbations and mortality in patients with chronic obstructive pulmonary disease. Thorax 2005;60:925-931.

11 Office for National Statistics. Statistical Bulletin: Cancer survival in England. London: ONS, April 2010. Available: http://www.statistics.gov.uk/pdfdir/can0410.pdf (accessed 19/05/2010).

12 Inzucchi SE. Management of Hyperglycemia in the Hospital Setting. N Engl J Med 2006;355:1903-11.

13 Kosiborod M, Rathore SS, Inzucchi SE, et al. Admission glucose and mortality in elderly patients hospitalized with acute myocardial infarction: implications for patients with and without recognized diabetes. Circulation 2005;111:3078-86.

14 Baker EH, Janaway CH, Philips BJ, Brennan AL, Baines DL, Wood DM, et al. Hyperglycaemia is associated with poor outcomes in patients admitted to hospital with acute exacerbations of chronic obstructive pulmonary disease. Thorax 2006;61:284–9.

15 Chakrabarti B, Angus RM, Agarwal S, Lane S, Calverley PMA. Hyperglycaemia as a predictor of outcome during non-invasive ventilation in decompensated COPD. Thorax 2009;64:857–862.

16 Moretti M, Cilione C, Tampieri A, Fracchia C, Marchioni A, Nava S. Incidence and causes of non-invasive mechanical ventilation failure after initial success. Thorax 2000; 55:819-825.

17 Louis M, Punjabi NM. Effects of acute intermittent hypoxia on glucose metabolism in awake healthy volunteers. J Appl Physiol. 2009;106:1538-44.

18 Adrogué HJ, Chap Z, Okuda Y, Michael L, Hartley C, Entman M, Field JB. Acidosis-induced glucose intolerance is not prevented by adrenergic blockade. Am J Physiol. 1988;255:E812-23.

19 Van Cromphaut SJ. Hyperglycaemia as part of the stress response: the underlying mechanisms. Best Pract Res Clin Anaesthesiol. 2009;23:375-86.

20 Slatore CG, Bryson CL, Au DH. The Association of Inhaled Corticosteroid Use with Serum Glucose Concentration in a Large Cohort. The American Journal of Medicine 2009;122;472-478

21 Blackburn D, Hux J, Mamdani M. Quantification of the risk of corticosteroid-induced diabetes mellitus among the elderly. J Gen Intern Med. 2002;17:717-720.

22 Van den Berghe G, Wouters P, Weekers F, et al. Intensive insulin therapy in critically ill patients. N Engl J Med 2001; 345:1359-67.

23 The NICE-SUGAR Study Investigators. Intensive versus conventional glucose control in critically ill patients. N Engl J Med 2009;360:1283-97.

24 Brunkhorst FM, Engel C, Bloos F, et al. Intensive insulin therapy and pentastarch resuscitation in severe sepsis. N Engl J Med 2008;358:125-39.

25 Anantharaman R, Heatley M, Weston CFM. Hyperglycaemia in acute coronary syndromes: risk-marker or therapeutic target? Heart 2009;95:697-703

26 Malmberg K, Ryden L, Efendic S, et al. Randomized trial of insulin-glucose infusion followed by subcutaneous insulin treatment in diabetic patients with acute myocardial infarction (DIGAMI study): effects on mortality at 1 year. J Am Coll Cardiol 1995;26:57–65.

27 Malmberg K, Ryden L, Wedel H, et al. Intense metabolic control by means of insulin in patients with diabetes mellitus and acute myocardial infarction (DIGAMI 2): effects on mortality and morbidity. Eur Heart J 2005;26:650–61.

28 The CREATE-ECLA Trial Group Investigators. Effect of glucose-insulin-potassium infusion on mortality in patients with acute ST-segment elevation myocardial infarction. JAMA 2005;293:437–46.

29 Scottish Intercollegiate Guidelines Network. SIGN 93. Acute coronary syndromes. A national clinical guideline. Available: http://www.sign.ac.uk/pdf/sign93.pdf (accessed 23/03/2010).

30 Deedwania P, Kosiborod M, Barrett E, et al. Hyperglycemia and acute coronary syndrome. A Scientific Statement from the American Heart Association Diabetes Committee of the Council on Nutrition, Physical Activity, and Metabolism. Circulation 2008;117:1610–9.

31 The Task Force on the management of ST-segment elevation acute myocardial infarction of the European Society of Cardiology. Eur Heart J 2008;29:2909–2945.

32 The Task Force for the Diagnosis and Treatment of Non-ST-Segment Elevation Acute Coronary Syndromes of the European Society of Cardiology. Guidelines for the diagnosis and treatment of non-ST-segment elevation acute coronary syndromes. Eur Heart J 2007;28:1598–1660

33 Moghissi ES, Korytkowski MT, DiNardo MT et al. American Association of Clinical Endocrinologists and American Diabetes Association Consensus Statement on Inpatient Glycemic Control. Diabetes Care 2009;32:1119–1131.

34 National Collaborating Centre for Chronic Conditions. Type 2 diabetes: national clinical guideline for management in primary and secondary care (update). London: Royal College of Physicians, 2008.

35 Bailey CJ, Turner RC. Metformin. N Engl J Med 1996;334:574–579.

36 TEVA UK Limited. Metformin 500 mg Tablets. Summary of Product Characteristics (Version 10). Available: http://www.tevauk.com/webroot/files/products/283/files/Metformin500mgTabletsPL00289_0340v10.pdf (accessed 12/07/2010).

37 Gore DC, Ferrando AA, Wolf SE, et al. Metformin blunts stress-induced hyperglycemia following thermal injury. J Trauma. 2003;54:555–561.

38 Gore DC. Wolf SE. Sanford A. Herndon DN. Wolfe RR. Influence of metformin on glucose intolerance and muscle catabolism following severe burn injury. Annals of Surgery 2005; 241(2):334-42.

39 Joint Formulary Committee. British National Formulary. 58th ed. London: British Medical Association and Royal Pharmaceutical Society of Great Britain; 2009

40 Lalau JD, Race JM. Lactic acidosis in metformin therapy: searching for a link with metformin in reports of `metformin-associated lactic acidosis'. Diabetes, Obesity and Metabolism 2001;3:195–201

41 Salpeter SR, Greyber E, Pasternak GA, Salpeter (posthumous) EE. Risk of fatal and nonfatal lactic acidosis with metformin use in type 2 diabetes mellitus. Cochrane Database of Systematic Reviews 2010, Issue 1. Art. No.: CD002967. DOI: 10.1002/14651858.CD002967.pub3.

42 Stumvoll M, Nurjhan N, Perriello G, Dailey G, Gerich JE. Metabolic effects of metformin in non-insulin-dependent diabetes mellitus. N Engl J Med 1995; 333:550–4.

43 Wollen N, Bailey CJ. Inhibition of hepatic gluconeogenesis by metformin: synergism with insulin. Biochem Pharmacol 1988;37:4353-8.
44 Rossetti L, DeFronzo RA, Gherzi R, et al. Effect of metformin treatment on insulin action in diabetic rats: in vivo and in vitro correlations. Metabolism 1990;39:425-35.
45 Shaw RJ, Lamia KA, Vasquez D, et al. The kinase LKB1 mediates glucose homeostasis in liver and therapeutic effects of metformin. Science 2005; 310:1642–6.
46 Stumvoll M, HäringH-U, Matthaei S. Metformin. Endocrine Research 2007;32:39–57.
47 Zhou G, Myers R, Li Y, et al. Role of AMP-activated protein kinase in mechanism of metformin action. J Clin Invest 2001; 108:1167–74.
48 Isoda K, Young JL, Zirlik A, MacFarlane LA, Tsuboi N, Gerdes N, Schonbeck U, Libby P. Metformin Inhibits Proinflammatory Responses and NuclearFactor-B in Human Vascular Wall Cells. Arterioscler Thromb Vasc Biol. 2006;26:611-617.
49 Huang NL, SH Chiang, CH Hsueh, YJ Liang, YJ Chen, LP Lai. Metformin inhibits TNF-α-induced IκB kinase phosphorylation, IκB-α degradation and IL-6 production in endothelial cells through PI3K-dependent AMPK phosphorylation. International Journal of Cardiology 2009;134:169–175.
50 Faure P, Rossini E, Wiernsperger N, et al: An insulin sensitizer improves the free radical defense system potential and insulin sensitivity in high fructose-fed rats. Diabetes 1999;48:353-357.
51 Bonnefont-Rousselot D, Raji B, Walrand S, et al. An intracellular modulation of free radical production could contribute to the beneficial effects of metformin towards oxidative stress. Metabolism 2003;52:586-589.
52 Formoso G. De Filippis EA. Michetti N. Di Fulvio P. Pandolfi A. Bucciarelli T. Ciabattoni G. Nicolucci A. Davi G. Consoli A. Decreased in vivo oxidative stress and decreased platelet activation following metformin treatment in newly diagnosed type 2 diabetic subjects. Diabetes/Metabolism Research Reviews 2008;24(3):231-7.
53 Johansen K. Efficacy of metformin in the treatment of NIDDM. Diabetes Care 1999;22:33–7.
54 Saenz A, Fernandez-Esteban I, Mataix A, Ausejo Segura M, Roqué i Figuls M, Moher D. Metformin monotherapy for type 2 diabetes mellitus. Cochrane Database of Systematic Reviews 2005, Issue 3. Art. No.: CD002966. DOI: 10.1002/14651858.CD002966.pub3.
55 UK Prospective Diabetes Study (UKPDS) Group. Effect of intensive blood-glucose control with metformin on complications in overweight patients with type 2 diabetes (UKPDS 34). Lancet 1998;352:854-65
56 Diabetes Prevention Program Research Group. Knowler WC. Fowler SE. Hamman RF. Christophi CA. Hoffman HJ. Brenneman AT. Brown-Friday JO. Goldberg R. Venditti E. Nathan DM. 10-year follow-up of diabetes incidence and weight loss in the Diabetes Prevention Program Outcomes Study. Lancet 2009; 374(9702):1677-86.
57 Bailey CJ. Metformin: effects on micro and macrovascular complications in type 2 diabetes mellitus. Cardiovasc Drugs Ther 2008;22:215-224.
58 Wiernsperger NF. Metformin: intrinsic vasculoprotective properties. Diabetes Technology and Theratpeutics 2000;2:259-272.
59 MacNee W. Pulmonary and systemic oxidant/antioxidant imbalance in chronic obstructive pulmonary disease. Proc Am Thorac Soc 2005;2:50–60.
60 Rahman I, Adcock IM. Oxidative stress and redox regulation of lung inflammation in COPD. Eur Respir J 2006; 28: 219–242
61 Kirkham P, Rahman I. Oxidative stress in asthma and COPD: Antioxidants as a therapeutic strategy. Pharmacology & Therapeutics 111 (2006) 476 – 494
62 Rahman I, Skwarska E, MacNee W. Attenuation of oxidant/antioxidant imbalance during treatment of exacerbations of chronic obstructive pulmonary disease. Thorax 1997;52:565–8.
63 Barnes PJ, Adcock IM. Glucocorticoid resistance in inflammatory diseases. Lancet 2009;373:1905–17.
64 Dykens JA, Jamieson J, Marroquin L, Nadanaciva S, Billis PA, Will Y. Biguanide-induced mitochondrial dysfunction yields increased lactate production and cytotoxicity of aerobically-poised HepG2 cells and human hepatocytes in vitro. Toxicology and Applied Pharmacology 2008;233:203–210.
65 Rachmani R, Slavachevski I, Levi Z, Zadok B-S, Kedar Y, Ravid M. Metformin in patients with type 2 diabetes mellitus: reconsideration of traditional contraindications. Eur J Int Med 2002;13:428–433.

66 Sulkin TV, Bosman D, Krentz AJ. Contraindications to metformin therapy in patients with NIDDM. Diabetes Care 1997;20:925–928

67 Sweileh WM. Contraindications to metformin therapy among patients with type 2 diabetes mellitus. Pharm World Sci 2007;29:587–592.

68 Misbin RI. The phantom of lactic acidosis due to metformin in patients with diabetes. Diabetes Care 2004;27:1791-1793.

69 Diabetes Prevention Program Outcomes Study Research Group. Protocol for the diabetes prevention program outcomes study (version 3.0). Available: http://www.bsc.gwu.edu/dpp/protocol.htmlvdoc (accessed 30/03/2010)

70 Malnutrition Universal Screening Tool (MUST). Redditch: British Association for Enteral and Parenteral Nutrition (BAPEN), 2008. Available: http://www.bapen.org.uk/musttoolkit.html (accessed 14/07/2010).

71 University of Leicester and the UK National Screening Committee. The handbook for vascular risk assessment, risk reduction and risk management (2008). Available: http://www.screening.nhs.uk/cms.php?folder=2718 (accessed 30/03/2010)

72 British Thoracic Society COPD Consortium. Spirometry in Practice (2nd edition, 2005). Available: http://www.brit-thoracic.org.uk/clinical-information/copd/copd-consortium-publications.aspx (accessed 30/03/2010)

73 Brown PH, Teelucksing S, Matusiewicz SP et al. Cutaneous vasoconstrictor response to glucocorticoids in asthma. Lancet 1991;337:576–580.

74 Livingston E, Chaudhuri R, McMahon AD, et al. Systemic sensitivity to corticosteroids in smokers with asthma. Eur Respir J 2007;29:64–70
