# Supplementary material for: Metformin in severe exacerbations of chronic obstructive pulmonary disease: a randomised controlled trial
Source: Thorax. 2016 Feb 25;71(7):587–93. doi: 10.1136/thoraxjnl-2015-208035 (PMC4941151; doi:10.1136/thoraxjnl-2015-208035)
Supplement: Supplementary tables [file thoraxjnl-2015-208035supp_tables.pdf]

## Supplementary Table 1

### *Concomitant respiratory medications during the in-hospital phase*

| Drug class | All | Metformin | Placebo | P-value |
|---|---|---|---|---|
| **Inhaled/nebulised therapy** | **(n=51*)** | **(n=33*)** | **(n=18)** | |
| Short-acting β-agonist | 49 (96%) | 31 (94%) | 18 (100%) | 0.287 |
| Short-acting antimuscarinic | 37 (73%) | 23 (70%) | 14 (78%) | 0.537 |
| Long-acting β-agonist (without ICS) | 3 (6%) | 2 (6%) | 1 (6%) | 0.942 |
| Long-acting antimuscarinic | 27 (53%) | 19 (58%) | 8 (44%) | 0.369 |
| ICS–LABA combination | 46 (90%) | 30 (91%) | 16 (89%) | 0.817 |
| **Systemic therapy** | **(n=49†)** | **(n=31†)** | **(n=18)** | |
| Systemic corticosteroid | 48 (98%) | 30 (97%) | 18 (100%) | 0.441 |
| Antibiotic | 43 (88%) | 29 (94%) | 14 (78%) | 0.105 |
| Methylxanthine | 17 (35%) | 8 (26%) | 9 (50%) | 0.086 |
| Mucolytic | 37 (76%) | 22 (71%) | 15 (83%) | 0.332 |
| Leukotriene receptor antagonist | 1 (2%) | 1 (3%) | 0 (0%) | 0.441 |
| Magnesium | 1 (2%) | 0 (0%) | 1 (6%) | 0.185 |

ICS, inhaled corticosteroid; LABA, long-acting β-agonist.

* Records of inhaled/nebulised medications were not available for one participant

† Records of acute systemic respiratory medications were not available for three participants

## Supplementary Table 2

### *Characteristics of the study groups with respect to severity of airflow limitation*

| Characteristic* | *Summary metric** | All (n=52) | Metformin (n=34) | Placebo (n=18) | P-value |
|---|---|---|---|---|---|
| **Spirometry not completed** | *n (%[i])* | 9 (17%) | 7 (21%) | 2 (11%) | 0.470 |
| **$FEV_1$:FVC ratio ≥0.7** | *n (%[ii])* | 5 (12%) | 4 (15%) | 1 (6%) | 0.642 |
| **$FEV_1$:FVC ratio <0.7** | *n (%[ii])* | 38 (88%) | 23 (85%) | 15 (94%) | |
| GOLD 1 (≥80% predicted) | *n (%[iii])* | 1 (3%) | 0 (0%) | 1 (7%) | 0.677 |
| GOLD 2 (50–79% predicted) | *n (%[iii])* | 10 (26%) | 6 (26%) | 4 (27%) | |
| GOLD 3 (30–49% predicted) | *n (%[iii])* | 15 (39%) | 10 (43%) | 5 (33%) | |
| GOLD 4 (<30% predicted) | *n (%[iii])* | 12 (32%) | 7 (30%) | 5 (33%) | |
| Best $FEV_1$† — % predicted | *mean±SD* | 44±25 | 43±16 | 47±35 | 0.699 |

$FEV_1$, forced expiratory volume in 1 second, expressed as a percentage of the predicted value; FVC, forced vital capacity; GOLD, Global initiative for chronic Obstructive Lung Disease; SD, standard deviation

* The denominators for calculation of percentages are: *i.* all participants; *ii.* all participants who undertook spirometry; *iii.* all participants with obstructive spirometry ($FEV_1$:FVC ratio <0.7)

† The 'best' $FEV_1$ is the highest value recorded from the valid spirometry attempts made at hospital discharge and 1-month follow-up, among patients with $FEV_1$:FVC ratio <0.7.

## Supplementary Table 3

### *Sensitivity analyses using alternative summary metrics of in-hospital blood glucose concentration*

| **Summary metric** | **Mean (±SD)** | | **Between-group difference (95% CI)** | **P-value** |
|---|---|---|---|---|
| | **Metformin group (n=34)** | **Placebo group (n=18)** | | |
| Mean pre-breakfast glucose concentration (mmol/L) | 5.0±0.8 | 5.5±2.3 | −0.6 (−1.4 to +0.3) | 0.197 |
| Peak capillary blood glucose concentration (mmol/L) | 12.4±3.3 | 12.4±4.8 | 0.05 (−2.2 to +2.3) | 0.966 |

SD, standard deviation; CI, confidence interval

## Supplementary Table 4

### *Sensitivity analysis using subgroups defined by baseline (day 1) blood glucose concentration*

| Subgroup | Mean ±SD | | Between-group difference (95% CI) | P-value |
|---|---|---|---|---|
| | Metformin group | Placebo group | | |
| Participants with mean day 1 blood glucose concentration in lower 50th centile | 6.8±0.6 (n=16) | 6.6±0.6 (n=10) | −0.1 (−0.4 to +0.6) | 0.604 |
| Participants with mean day 1 blood glucose concentration in upper 50th centile | 7.3±1.0 (n=18) | 9.6±4.6 (n=8) | −2.3 (−6.1 to +1.5) | 0.201 |
| Participants with mean day 1 blood glucose concentration in upper 50th centile (excluding outlying observation*) | 7.3±1.0 (n=18) | 8.1±1.7 (n=7) | −0.8 (−2.4 to +0.8) | 0.166 |

SD, standard deviation; CI, confidence interval

*Outlying observation in the placebo group with a mean blood glucose concentration more than 10 standard deviations from the overall cohort mean; see manuscript for further details.
