# Supplementary material for: Metformin in severe exacerbations of chronic obstructive pulmonary disease: a randomised controlled trial
Source: Thorax. 2016 Feb 25;71(7):587–93. doi: 10.1136/thoraxjnl-2015-208035 (PMC4941151; doi:10.1136/thoraxjnl-2015-208035)

Supplementary Figure 1. Mean (standard deviation) capillary blood glucose concentrations for the first 7 inpatient study days.

Day 1 was defined as the first day on which the study medication was administered. Panel A includes all patients in the study. In Panel B, one outlier, whose mean glucose was more than ten standard deviations from the cohort mean, has been excluded from the placebo group in accordance with plans for a sensitivity analysis made after data collection but before unblinding. There was no significant interaction between treatment allocation and day of treatment in either case (P=0.832 and 0.618 in Panels A and B, respectively, ANOVA).

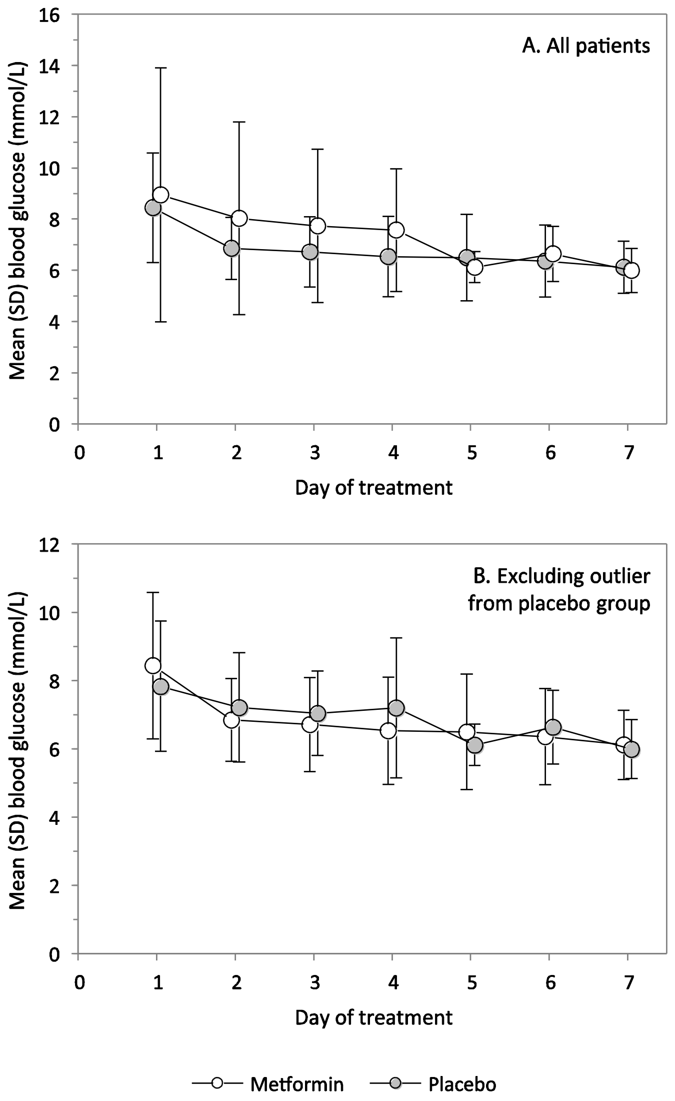

Supplement: Supplementary figure — Mean (standard deviation) capillary blood glucose concentrations for the first 7 inpatient study days. [file thoraxjnl-2015-208035supp_figure.pdf]
